# Video Transformer for Segmentation of Echocardiography Images in Myocardial Strain Measurement

**DOI:** 10.1007/s10278-025-01682-5

**Published:** 2025-09-17

**Authors:** Kuan-Chih Huang, Chang-En Lin, Donna Shu-Han Lin, Ting‐Tse Lin, Cho-Kai Wu, Geng-Shi Jeng, Lian-Yu Lin, Lung-Chun Lin

**Affiliations:** 1 Graduate Institute of Clinical Medicine, College of Medicine, National Taiwan University, Taipei, Taiwan; 2 National Taiwan University Hospital, Hsin-Chu Branch, Hsinchu, Taiwan; 3 Section of Cardiology, Department of Internal Medicine, National Taiwan University Hospital, No. 7, Chung-Shan S. Road, Taipei, 100225 Taiwan; 4 Division of Cardiology, Department of Internal Medicine, Su Memorial Hospital, Shin Kong Wu Ho, Taipei, Taiwan; 5 Institute of Electronics, National Yang Ming Chiao Tung University, Hsinchu, Taiwan

**Keywords:** Left ventricular global longitudinal strain, Precision, Transformer, Temporal consistency

## Abstract

The adoption of left ventricular global longitudinal strain (LVGLS) is still restricted by variability among various vendors and observers, despite advancements from tissue Doppler to speckle tracking imaging, machine learning, and, more recently, convolutional neural network (CNN)-based segmentation strain analysis. While CNNs have enabled fully automated strain measurement, they are inherently constrained by restricted receptive fields and a lack of temporal consistency. Transformer-based networks have emerged as a powerful alternative in medical imaging, offering enhanced global attention. Among these, the Video Swin Transformer (V-SwinT) architecture, with its 3D-shifted windows and locality inductive bias, is particularly well suited for ultrasound imaging, providing temporal consistency while optimizing computational efficiency. In this study, we propose the DTHR-SegStrain model based on a V-SwinT backbone. This model incorporates contour regression and utilizes an FCN-style multiscale feature fusion. As a result, it can generate accurate and temporally consistent left ventricle (LV) contours, allowing for direct calculation of myocardial strain without the need for conversion from segmentation to contours or any additional postprocessing. Compared to EchoNet-dynamic and Unity-GLS, DTHR-SegStrain showed greater efficiency, reliability, and validity in LVGLS measurements. Furthermore, the hybridization experiments assessed the interaction between segmentation models and strain algorithms, reinforcing that consistent segmentation contours over time can simplify strain calculations and decrease measurement variability. These findings emphasize the potential of V-SwinT-based frameworks to enhance the standardization and clinical applicability of LVGLS assessments.

**Supplementary Information:**

The online version contains supplementary material available at 10.1007/s10278-025-01682-5.

## Introduction

The progression of heart failure with preserved ejection fraction (HFpEF) is theorized to follow a sequence where left ventricular global longitudinal strain (LVGLS) declines initially, followed by compensatory enhancement of left ventricular global circumferential strain (LVGCS) to sustain a preserved ejection fraction [1]. However, the measurement of LVGLS is subject to three primary sources of error [2]. First, observer-specific variability arises from differences in selecting keyframes (end-diastole, ED; end-systole ES) and segmentation of myocardial contours. Second, vendor-specific differences in ultrasonic speckle-tracking methods and proprietary motion estimation algorithms can lead to inconsistencies in strain calculations across various ultrasound systems. Third, additional variability is introduced by vendor-specific regularization and strain calculation algorithms [3]. Machine learning techniques and generalized left ventricular (LV) contour modeling have been developed to enhance consistency. However, experts’ manual adjustments often conflict with the software’s automated modeling. Even when these manual corrections are accepted, subsequent automated processes often struggle to align with the adjustments.

The next paradigm shift in LVGLS computation is expected to involve artificial intelligence (AI)-based segmentation, enabling the direct measurement of myocardial length. The success of left ventricular ejection fraction (LVEF) estimation via LV segmentation [4] spurred subsequent tasks of LVGLS calculation based on LV segmentation with a convolutional neural network (CNN) architecture [5–7]. However, CNN-based segmentation has notable limitations. The restricted perception field and lack of temporal consistency can lead to unsatisfactory LV segmentation, which may cause oscillations in strain-to-time curves and further introduce errors in the timing and values of end-diastolic (ED) and end-systolic (ES) points. Although the In Defense of Online Models for Video Instance Segmentation (IDOL) can provide better temporal consistency in the task of LV segmentation strain with transformer-based attention mechanisms and contrastive learning for online video instance segmentation [8, 9], the CNN-based feature extraction is still prone to incomplete, fragmented, or wrong LV contour segmentation in specific scenarios, such as apical hypertrophic cardiomyopathy, regional wall motion abnormalities (RWMA), or with suboptimal images.

Recently, transformers have shown promising results in medical image segmentation. The Vision Transformer ViT [10] utilizes multi-head self-attention to perform feature extraction, thereby enhancing long-range dependency. This capability is particularly advantageous for processing cardiac ultrasound images, which are inherently unconstrained and subject to shifts, rotations, scaling, and patient-related factors such as movement or anatomical variability. SETR [11] was one of the first models to apply the ViT framework to semantic segmentation. It introduced the concept of a sequence-to-sequence Transformer architecture, showing that Transformers could indeed be effective in pixel-level predictions. However, it was limited by high computational demands. SegFormer [12] incorporated a positional-encoding-free, hierarchical Transformer encoder with a lightweight MLP-based decoder. Such a multi-scale feature extraction approach ensures high efficiency and robustness in segmentation tasks while reducing computational costs. Swin Transformer (SwinT) [13] introduced shifted window-based attention and a hierarchical structure that enabled its multi-scale feature representation and spatial locality. With compatible decoders such as FPN and U-net, SwinT can perform semantic or even instance segmentation, which is more suitable for medical imaging analysis. Liao et al. [14] adopted SwinT + K-Net and SegFormer to perform LV segmentation at ED and ES frames, and they reported that the performance of these transformer-based models outperformed traditional DeepLab v3 +, the TransBridge model, and the Trans U-Net model. They also reported a high dice score of the SwinT-based model even without post-processing. Video Swin Transformer (V-SwinT) [15] further extends conventional 2D self-attention mechanisms into the spatiotemporal domain through 3D window-based multi-head self-attention (3D W-MSA), allowing the model to jointly capture intra-frame spatial relationships and inter-frame temporal dynamics. This is particularly advantageous for echocardiographic strain analysis, where accurate tracking of myocardial motion over time is essential. By using 3D shifted windows, V-SwinT adds a locality inductive bias that enhances temporal and spatial feature extraction without the computational cost of global attention. Additionally, 3D relative position bias is incorporated to further refine the model’s ability to learn spatial and temporal positioning across frames, enabling precise modeling of cardiac dynamics in video sequences. The 3D shifted window mechanism also facilitates information flow across adjacent temporal segments, which promotes smoother contour evolution and reduces noise-induced fluctuations in strain–time curves. Furthermore, V-SwinT’s hierarchical structure supports multi-scale convolutional fusion, enabling the network to preserve fine-grained local details while maintaining global structural coherence—critical for accurate segmentation under suboptimal imaging or in the presence of pathology (e.g., regional wall motion abnormality or hypertrophic cardiomyopathy). These architectural features collectively make V-SwinT well suited for dynamic modeling tasks in echocardiography.

To overcome the aforementioned challenges, we proposed the DTHR-SegStrain (dynamic transformer with high resolution, applying segmentation for strain measurement) based on the V-SwinT model for LV myocardial segmentation and adopted the design of multi-scale feature fusion from FCN [16] and a contour regressor for further strain calculation algorithm. Importantly, our approach intentionally decouples the two core components of AI-based strain analysis—myocardial segmentation and strain computation—to enable independent evaluation and flexible adaptation. By benchmarking against existing strain estimation pipelines [5, 7] and exploring different combinations of segmentation and calculation modules, we aim to gain deeper insights into the relative contribution of each component and the potential for model hybridization across domains.

## Methods

### Training Materials for VSwinT-Based LV Segmentation

The training dataset for LV segmentation was carefully curated to ensure diversity and comprehensive coverage. It consists of 2340 anonymized echocardiograms, including 1282 videos from Taiwanese institutions, 72 videos featuring athletes from the Check-up Your Heart Program of the International University Sports Federation (FISU) during the 2017 Summer Universiade in Taipei [17], and 986 videos from the publicly available CAMUS dataset [18]. These 2340 videos featured a diverse distribution of imaging systems, including Philips Medical Systems iE33 (38.12%) and EPIQ 7 C (16.41%), GE Vivid systems (2.68% collectively, FISU), and GE Vivid E95 machines (42.14%, CAMUS). As a result, the model was exposed to a wide range of imaging conditions, types of machines, and patient demographics, which enhances its robustness and adaptability to clinical variability.

Similar to CAMUS annotation, a multitask annotation strategy was performed frame-by-frame on the 1282 institutional and 72 FISU videos by two expert cardiologists—the first and corresponding authors of this study—both of whom fulfill the Level III-IE competency criteria as defined by the American Society of Echocardiography. In addition to training data annotation, these same cardiologists also manually annotated and measured strain in a separate 1050-case validation dataset. While CAMUS annotated only the systolic phase, we performed full-length annotation of two cardiac cycles in each video. This included annotations of the LV endocardial line to define the inner boundary of the LV for assessing ventricular volume and function, the LV epicardial line to outline the outer boundary for measuring myocardial thickness and wall motion, and the LA endocardial line to mark the inner boundary of the LA for evaluating atrial size and function.

The pre-processing pipeline converts DICOM frames to 8-bit grayscale images and normalizes pixel values to a range of 0 to 255. Each frame is cropped to square dimensions based on the smaller of width and height, resized to 224 × 224 pixels, and expanded into three channels before being normalized to a range of 0 to 1. Videos are segmented into chunks of 64 frames with a 10-frame overlap, ensuring consistency in temporal resolution. For videos with fewer than 64 frames, the final frame is repeated to meet the required length. The resulting frames are stacked into tensors of shape [64, 224, 224, 3] and transposed to [3, 64, 224, 224] to align with the transformer’s input specifications. All chunks are aggregated into tensors with a final shape of [#Chunk, 3, 64, 224, 224].

The model outputs the 2D coordinates of 49 LV endocardial contour points for each frame. This facilitates detailed tracking of left ventricular shape and function across the cardiac cycle, enabling precise contour-based assessments of myocardial performance.

Key training parameters were carefully selected to optimize performance. The loss function utilized was the mean squared error (MSE), calculated for each coordinate to minimize regression errors. The Adam optimizer was employed with a learning rate of 0.0001, a momentum of 0.9, and a weight decay of 0.0001, ensuring stable convergence during training. Extensive data augmentation techniques were implemented to improve the model’s robustness and generalizability. These techniques included brightness adjustments with a variance of 75 and contrast scaling by a factor of 0.3. Additionally, geometric transformations were applied, such as shifts of up to 6.25%, scaling by up to 20%, and rotations within a range of ± 40°.

### *Model Architecture (*Fig. 1*)*

**Fig. 1:**
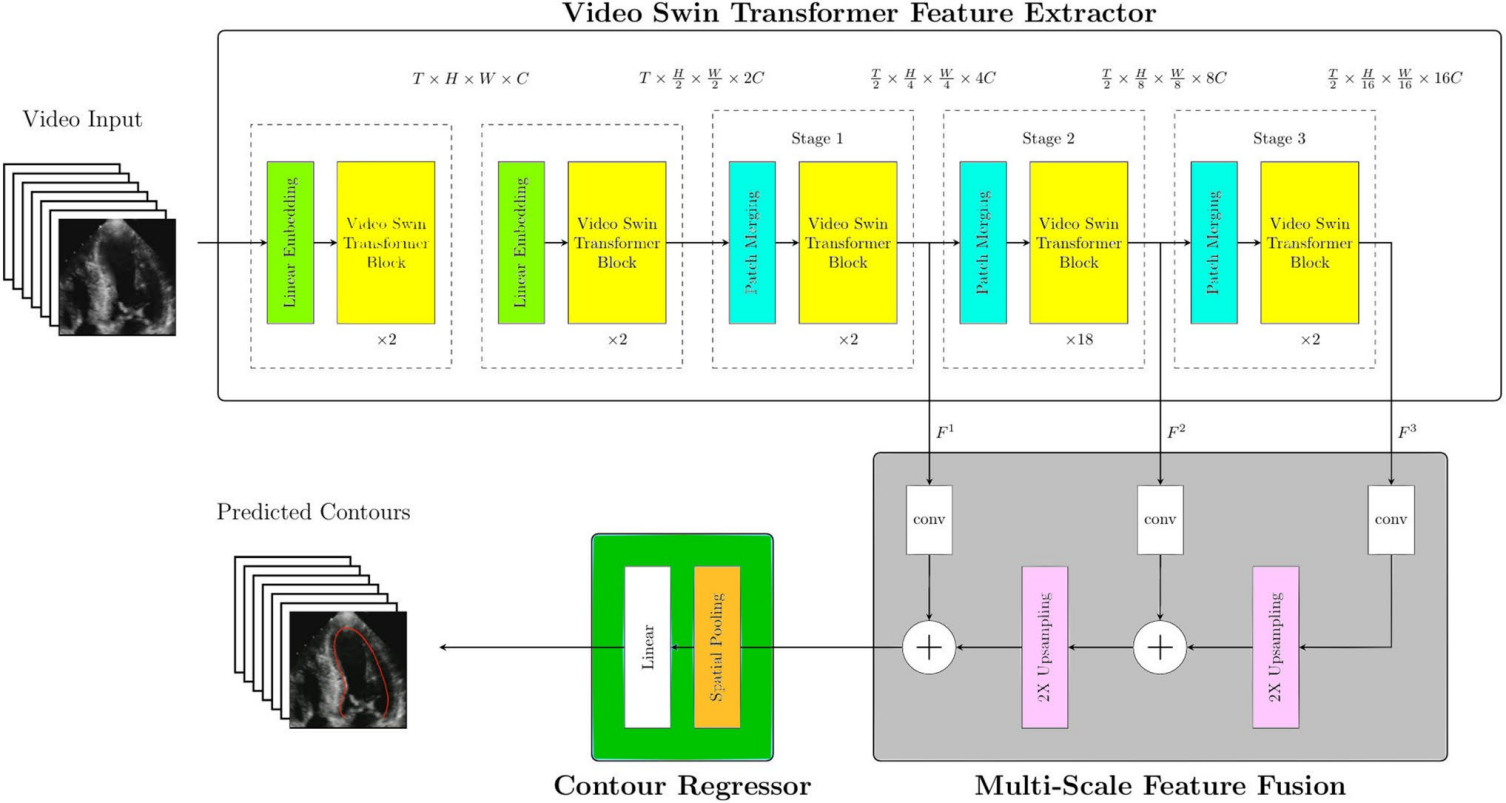
The network architecture of the proposed DTHR-SegStrain. The DTHR-SegStrain is comprised of three phases: a the Video Swin Transformer feature extractor, b an FCN-style multiscale feature fusion, and c a contour regressor. Therefore, instead of a segmentation mask or a heatmap for key points, the model outputs the 2D coordinates of 49 LV endocardial contour points for each frame. These coordinates are adopted for myocardial length calculation without further postprocessing

The DTHR-SegStrain model integrates a V-SwinT backbone for spatiotemporal feature extraction and a contour regression network for precise 2D contour point prediction of the LV endocardium from echocardiogram videos. The architecture is carefully designed to leverage multi-scale representations and hierarchical attention mechanisms to process medical video data efficiently.

### Video Swin Transformer (V-SwinT) Backbone

In this study, we adopted the original design of the V-SwinT framework for our LV segmentation model. The V-SwinT backbone comprises three sequential stages (Stage 1–Stage 3) (Fig. 1).

- Within each stage, a stack of Transformer blocks is applied. Every block first performs 3-D Windowed Multi-Head Self-Attention (3D W-MSA) over a local cuboid of $8\times 7\times 7$ tokens, followed by a position-wise feed-forward network. The next block in the pair repeats the same operations after shifting the attention window by $(4,3,3)$ tokens along the temporal, height, and width axes, respectively. This standard-window → shifted-window alternation allows tokens that were previously separated by the window boundary to attend to one another, thereby eliminating spatiotemporal seams and enlarging the receptive field linearly with network depth.
- After the ending block of the last 3 stages, a spatial 2 × 2 patch-merging layer halves the height and width while preserving the full temporal length.
- The encoder therefore emits three temporally intact feature tensors that will later be fused:
  $${F}_{1}=32\times 28\times 28,{F}_{2}=32\times 14\times 14,{F}_{3}=32\times 7\times 7.$$

The following paragraphs explain the architectural ingredients that help the V-SwinT encoder to model spatiotemporal features, enhance temporal consistency, and merge multi-scale features that capture local and global semantic information:

- **Two-frame spatiotemporal tokenization**
  The input clip $(T\times H\times W\times C)$ is partitioned into cubes of $\left({t}_{p},{h}_{p},{w}_{p}\right)=(2,4,4)$. Flattening and linearly projecting each 2 × 4 × 4 cube produces tokens that already encode two frames of wall motion, embedding short-range temporal context at the network input.
- **3-D Windowed MSA (3D W-MSA): local spatiotemporal modeling**
  Inside each $8\times 7\times 7(the original VSwinT setting)$ token window, multi-head self-attention jointly processes space and time:
  $$Attn\left(Q, K, V\right)=Softmax\left(\frac{{QK}^{{\top }}}{\sqrt{{d}_{k}}}+B\right)V$$
  where *B* represents the relative position bias matrix.
  This delivers explicit spatiotemporal features with $O(T)$ complexity in sequence length.
- **3-D shifted windows: enforcing temporal consistency**
  The second block of every pair shifts the window origin by $\left(\left[8/2\right],\left[7/2\right],\left[7/2\right]\right)=\left(4, 3, 3\right)$ tokens before re-applying 3D W-MSA. Tokens that were formerly separated by a window boundary can now attend to one another, which removes seams between successive cardiac phases, and expands the effective temporal receptive field to $\approx 4L$ frames after $L$ alternating blocks—sufficient to cover a full diastole–systole cycle in a 32-frame chunk. Thus the shifted-window design realizes temporal consistency without quadratic cost.
- **Time-preserving three-level hierarchy**
  After every two transformer stages, a spatial $2\times 2$ patch-merging layer halves resolution and doubles channel width while leaving the temporal dimension unchanged. The backbone therefore outputs the three tensors ${F}_{1},{F}_{2},{F}_{3}$ defined above, which jointly capture fine-scale wall motion (${F}_{1}$), mid-level anatomy (${F}_{2}$), and global chamber geometry (${F}_{3}$).

### Multi-Scale Feature Fusion

Because 3D W-MSA has already injected local temporal correlations, and shifted windows have propagated them across window borders; the fusion network can simply upsample $\left({F}_{2},{F}_{3}\right)$ to the resolution of ${F}_{1}$ and combine the three tensors with lightweight 2-D convolutions. This fully makes use of both local detail and global semantics.

The multi-scale feature maps ${{\boldsymbol{F}}}^{1},{{\boldsymbol{F}}}^{2},{{\boldsymbol{F}}}^{3}$ from the three stages of the V-SwinT backbone are fused using a strategy inspired by the FCN [16] architecture. This method progressively upsamples and merges multi-scale feature maps, preserving spatial details while integrating semantic context, effectively combining local and global information.

### Fusion Process

1. **Initial feature map resolutions**
  The smallest feature map ${{\boldsymbol{F}}}^{3}$ has height and width dimensions of $\left(\mathrm{7,7}\right)$.
  The intermediate feature map ${{\boldsymbol{F}}}^{2}$ has dimensions $\text{of }\left(14,14\right)$.
  The largest feature map ${{\boldsymbol{F}}}^{1}$ has dimensions $\text{of }\left(28,28\right)$.
2. **2D convolution:**
  Each feature map ${{\boldsymbol{F}}}^{{\boldsymbol{l}}}$ undergoes a spatial refinement and channel unification using a 2D convolution operation. This step ensures that all feature maps have the same channel dimension $D$ (here we choose $D$=1024), facilitating the subsequent upscaling and addition process:
  $${{\boldsymbol{F}}}^{{{\boldsymbol{l}}}{\prime}}={\mathrm{Conv2D}}\left({{\boldsymbol{F}}}^{{\boldsymbol{l}}}\right),\hspace{1em}{{\boldsymbol{F}}}^{{{\boldsymbol{l}}}{\prime}}\in {R}^{{H}_{l}\times {W}_{l}\times D}$$
3. **Progressive upscaling and addition:**
  The smallest feature map ${F}^{3{^{\prime}}}$ is scaled up by a factor of 2 to match the resolution of ${{\boldsymbol{F}}}^{2}$:
  $${F}_{\mathrm{scaled}}^{3^{\prime}} = {\mathrm{Upsample}}\left({F}^{3{^{\prime}}}\right)$$
  The upscaled ${{\boldsymbol{F}}}_{\mathrm{scaled}}^{3}$ is added to the intermediate feature map ${\mathbf{F}}^{2\boldsymbol{^{\prime}}}$:
  $${{\boldsymbol{F}}}^{2+}={{\boldsymbol{F}}}_{\mathrm{scaled}}^{3\boldsymbol{^{\prime}}}+{\mathbf{F}}^{2\boldsymbol{^{\prime}}}$$
  The combined feature map ${{\boldsymbol{F}}}^{2+}$ is further scaled up by a factor of 2 to match the resolution of ${\mathbf{F}}^{1\boldsymbol{^{\prime}}}$:
  $${{\boldsymbol{F}}}_{\mathrm{scaled}}^{2+}={\mathrm{Upsample}}\left({{\boldsymbol{F}}}^{2+}\right)$$
  Finally, ${{\boldsymbol{F}}}_{\mathrm{scaled}}^{2\boldsymbol{^{\prime}}+}$ is added to ${{\boldsymbol{F}}}^{{1}{\prime}}$:
  $${{\boldsymbol{F}}}_{\mathrm{fused}}={{\boldsymbol{F}}}_{\mathrm{scaled}}^{2+}+{{\boldsymbol{F}}}^{{1}{\prime}}$$
4. **Output: The resulting fused feature map **${{\boldsymbol{F}}}_{{\boldsymbol{f}}{\boldsymbol{u}}{\boldsymbol{s}}{\boldsymbol{e}}{\boldsymbol{d}}}$** integrates information from all scales:**
  $${{\boldsymbol{F}}}_{\mathrm{fused}} \in {R}^{{H}_{f} \times {W}_{f} \times D}$$

### Contour Regression Network

The fused feature map ${{\boldsymbol{F}}}_{{\boldsymbol{t}}}$ for each frame ${\boldsymbol{t}}$ is fed into the Contour Regression Network, which predicts the 2D coordinates of 49 contour points on the LV endocardium.

- **Global spatial pooling**
  To reduce spatial complexity, global pooling is applied to the fused feature map:
  $${{\boldsymbol{z}}}_{{\boldsymbol{t}}}={\mathrm{GlobalPool}}\left({{\boldsymbol{F}}}_{{\boldsymbol{t}}}\right)\in {R}^{D}$$
- **Coordinate prediction via fully connected linear layer**
  The pooled feature vector $,{{\boldsymbol{z}}}_{{\boldsymbol{t}}}$ is directly passed through a fully connected linear layer to predict the 2D coordinates of the 49 contour points, resulting in total 49 coordinates:
  $${{\boldsymbol{Y}}}_{{\boldsymbol{t}}}={\mathrm{Linear}}\left({{\boldsymbol{z}}}_{{\boldsymbol{t}}}\right)\in {R}^{49}$$
- **Final output**
  The final output is a sequence of 2D contour points for all frames:
  $$[{{\boldsymbol{Y}}}_{{\boldsymbol{t}}}{]}_{t=1}^{T}$$
- **Objective function**
  The model is trained using a mean squared error (MSE) loss between the predicted and ground-truth contour points:
  $$\mathcal{L}=\frac{1}{T}\sum\limits_{t=1}^{T}\frac{1}{49}\sum\limits_{i=1}^{49}{\left|\left|{{\boldsymbol{y}}}_{{\boldsymbol{t}},{\boldsymbol{i}}}-{\widehat{{\boldsymbol{y}}}}_{{\boldsymbol{t}},{\boldsymbol{i}}}\right|\right|}_{2}^{2}$$
  where ${{\boldsymbol{y}}}_{{\boldsymbol{t}},{\boldsymbol{i}}}$ and ${\widehat{{\boldsymbol{y}}}}_{{\boldsymbol{t}},{\boldsymbol{i}}}$ are the ground-truth and predicted 2 d coordinates of the ${\boldsymbol{i}}$-th point at frame ${\boldsymbol{t}}$.

### Profiling Highlights: The Strength of DTHR-SegStrain

Table 1 highlights the computational and resource characteristics of three models—Unity-GLS, EchoNet-dynamic, and DTHR-SegStrain—each tailored for specific use cases. Among these, DTHR-SegStrain demonstrates its superiority in video processing with remarkable efficiency and scalability. It processes 64 frames at a resolution of 224 × 224 with a total computational cost of 574.53 GFLOPs, which translates to only 8.98 GFLOPs per frame.

**Table 1:** Computational and resource characteristics of three segmentation models

MODEL	#Frame/batch	Input resolution	GFLOPs	GFLOPs/frame	#Parameters (M)	Minimum required GPU memory (MB)
Unity-GLS	1 (static)	608 × 608	275.77	275.77	66.97	499.85
EchoNet-Dynamic	1 (static)	112 × 112	7.83	7.83	39.63	162.22
DTHR-SegStrain	64 (video)	224 × 224	574.53	8.98	121.38	2289.51

*GFLOPs* giga floating-point operations per second, *M* mega, *MB* megabyte

With 121.38 million parameters, DTHR-SegStrain offers the most robust representational capacity among the three models. This supports complex feature extraction and advanced temporal modeling, positioning DTHR-SegStrain as an ideal choice for high-demand video understanding tasks. Furthermore, despite simultaneously processing 64 frames, DTHR-SegStrain efficiently manages its memory usage, requiring only 2289.51 MB of GPU memory. This balance between computational demands and memory consumption makes it well-suited for high-throughput video pipelines.

Comparatively, Unity-GLS excels in high-resolution spatial processing with its 608 × 608 input size, but its computational cost of 275.77 GFLOPs per frame limits its scalability for larger datasets or real-time applications. Conversely, EchoNet-dynamic is designed as a lightweight model, with a minimal cost of 7.83 GFLOPs per frame and a memory requirement of only 162.22 MB. While it is ideal for low-resolution or resource-constrained settings, it lacks the representational capacity and scalability required for more complex video tasks.

### Dynamic Programming Algorithm for ES/ES

The dynamic programming-based ED/ES detection algorithm identifies pairs of ED and ES frames from LV endocardial length time series extracted from echocardiographic videos. The algorithm optimally detects transitions between diastole and systole by maximizing a cost function that balances phase transition penalties and significant LV length changes. This process ensures accurate cardiac cycle detection and strain calculation for assessing myocardial function. For the details of the dynamic programming algorithm for ED/ES, please see supplementary materials.

### Study Population—Validation Dataset

A separate validation cohort of 1050 apical four-chamber (A4C) echocardiographic videos was consecutively collected from patients who underwent standard echocardiography. Each of the 1050 validation videos included manual strain measurements and left ventricular (LV) endocardial contour annotations at ED and ES. Both the contour labeling and LVGLS analysis were performed by two experienced cardiologists in the echocardiography lab using dedicated software (AutoStrain module, TTA 2.30, TomTec Imaging Systems GmbH, Unterschleissheim, Germany). For each case, the manually delineated contours—encompassing both endocardial and epicardial borders—were used to define the LV myocardial area and served as the reference for evaluating segmentation performance based on Dice coefficient, Intersection over Union (IoU), Hausdorff distance, and average Hausdorff distance. The manually measured strain values were used to establish the reference LVGLS against which the three AI-based models were compared. All data collection was approved by the institutional review board of our hospital (NTUH-REC no. 201809013RINB). Patient consent was waived due to the retrospective nature of the study and data anonymization.

### AI-Based Strain Calculation, a Hybridization Experiment

**Fig. 2:**
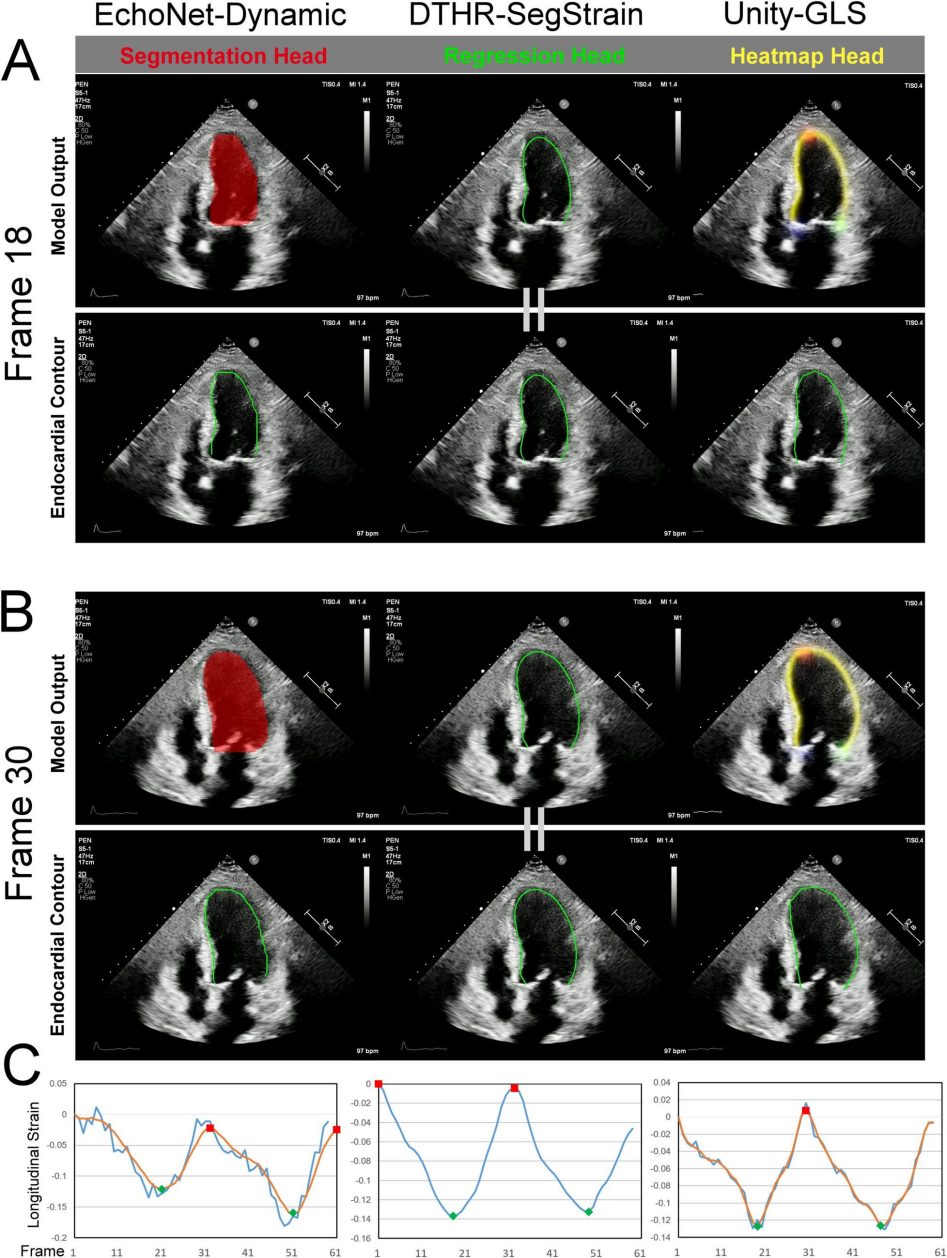
Three AI-assisted LV segmentation with proprietary strain algorithm. **A** End-systolic phase; **B** end-diastolic phase. The EchoNet-Dynamic outputs the LV segmentation mask (red mask), which is then transformed into the irregular, unsmooth contour border (**A, B**, bottom-left panels). The Unity-GLS generates four heatmaps, including three key points (**A, B**, up-right panels: red shadow, LV apex; blue shadow, basal septal hinge of the mitral valve; green shadow, basal lateral hinge of the mitral valve) and a zone for the LV endocardial contour (the yellow stripe). Subsequently, it predicts the LV endocardial contour that passes through the three key points while aligning as closely as possible with the brightest regions in the endocardial heatmap. Our DTHR-SegStrain directly yields endocardial contour via contour regressor (**A**&**B**, middle panels. The equal sign between the model output and the LV contour indicates that our model directly outputs the contour coordinates from the beginning). **C** Strain algorithms yield original strain-to-time curves (blue curves), apply smoothing filters (orange curves), and define end-diastolic (red squares) and end-systolic (green diamonds) time points. It is worth noting that only EchoNet (**C**, left) and Unity (**C**, right) apply a smoothing filter; no smoothing filter is adopted in DTHR-SegStrain (**C**, middle). Please refer to video S1 for dynamic visualization

LV segmentation models are commonly paired with proprietary strain calculation algorithms for AI-based LVGLS calculations (Fig. 2). To explore the interaction between LV contour prediction and strain calculation algorithms, a hybrid experimental setup was designed by combining three segmentation models with three strain calculation algorithms, resulting in nine distinct configurations. The first strain calculation method, referred to as the DTHR-SegStrain algorithm, is our proposed approach. This method leverages dynamic programming to identify the globally optimal ED and ES phases without requiring parameter tuning. This feature enhances its robustness against noise, ensuring reliable performance across varying conditions.

The second approach, the EchoNet-dynamic strain calculation method, utilizes the LV endocardial mask predicted by the EchoNet-dynamic model. The mask is dilated and transformed into an LV endocardial contour, from which a length time series is derived. Using SciPy’s find_peaks function, local maxima and minima are identified to pinpoint the ED and ES phases. This process, however, requires parameter tuning to optimize performance. Strain values are then calculated by pairing the ED and ES points and averaging the strain across these pairs.

The third method, Unity-GLS strain calculation, generates the LV endocardial heatmap via HRNet. The heatmap is converted into an LV endocardial contour, and a length time series is extracted. Like the EchoNet-dynamic method, this approach employs SciPy’s find_peaks function to detect ED and ES phases, requiring parameter tuning during the process. Strain is estimated by averaging the lengths at ED and ES phases separately and computing the strain based on these average values.

To enhance readability when describing the hybridization process, we simplify model names. For instance, “EchoNet-Dynamic LV contour + DTHR-SegStrain strain algorithm” is abbreviated as “EchoNet + DTHR,” where the part before the “ + ” represents the contour generation model, and the part after the “ + ” denotes the strain algorithm.

### Statistical Analysis

The results of GLS values were reported as mean ± SD. Compared to the expert’s strain values, Spearman’s coefficient, the intraclass correlation coefficient (ICC) and the Bland–Altman plot of each model were evaluated for each of the AI-assisted strain results. The ICC estimates were based on two-way mixed-effects models, with random effects for subjects and fixed effects for raters or methods, assessing absolute agreement in a single-rater or single-measurement context [19]. Receiver operating characteristic (ROC) analysis was conducted to assess each AI model’s ability to identify abnormal LVGLS values, defined as expert’s strain values outside the reference range (− 16% to − 18%) [20]. All statistical analyses were performed using STATA version 13 (StataCorp., College Station, Texas). All reported p values were 2-tailed, and *p* values < 0.05 were considered statistically significant.

## Results

### Characteristics of 1050 Clinical A4C Echocardiograms

A4C echocardiographic videos of 674 men and 376 women were retrospectively collected and summarized in Table 2.

**Table 2:** Characteristics of 1050 clinical A4C echocardiograms

	Total	Men	Women	*p*
n	1050	674	376	
Age	60.6 ± 13.5	62.8 ± 12.4	56.7 ± 14.5	< 0.01
Body height (cm)	163.8 ± 8.2	167.8 ± 6.5	156.8 ± 5.9	< 0.01
Body weight (kg)	66.7 ± 13.0	71.1 ± 11.7	59.1 ± 11.6	< 0.01
IVSd (mm)	10.2 ± 1.7	10.6 ± 1.8	9.6 ± 1.5	< 0.01
LVPWd (mm)	10.2 ± 4.7	10.6 ± 5.2	9.5 ± 3.6	< 0.01
LVIDd (mm)	52.0 ± 8.1	54.9 ± 7.7	47.0 ± 6.1	< 0.01
LVIDs (mm)	36.5 ± 10.3	40.1 ± 9.7	29.9 ± 7.8	< 0.01
LVEF (%)	55.9 ± 15.9	50.8 ± 14.8	65.1 ± 13.5	< 0.01
LAd (mm)	37.3 ± 6.0	39.0 ± 5.5	34.4 ± 5.6	< 0.01
LV mass	203.4 ± 70.2	229.0 ± 66.5	157.6 ± 50.8	< 0.01
LVGLS (%)	−16.5 ± 5.3	−15.6 ± 5.3	−18.1 ± 5.1	< 0.01

*LAd* left atrium diameter, *LVEF* left ventricular ejection fraction, *LVGLS* left ventricular global longitudinal strain, *IVSd* interventricular septal thickness, *LVIDd* left ventricular end diastole internal diameter, *LVIDs* left ventricular end-systole internal diameter, *LVPWd* left ventricular end diastole posterior wall thickness

### The Segmentation Performance of EchoNet, Unity, and V-SwinT

Among 1050 cases, EchoNet-dynamic failed LV contour segmentation task in 16 (1.5%) cases, Unity-GLS and DTHR-SegStrain can successfully complete all the tasks. The strain calculation algorithm of DTHR-SegStrain and EchoNet-Dynamic can complete the strain calculation from all kinds of LV segmentation contours, but the Unity-GLS failed 38 of 1050 V-SwinT contour, 43 of 1050 Unity-GLS contour, and 16 of 1038 of EchoNet-dynamic contour. Table 3 summarized the dice score, IoU, Hausdorff distance, and the average Hausdorff distance for the task of LV contour segmentation. The performances of DTHR-SegStrain (0.9073, 0.8341, 17.1316, 7.1883) and Unity-GLS (0.9097, 0.8410, 19.3001, 7.5315) were compatible and significantly outperformed those of EchoNet-dynamic (0.8306, 0.7172, 37.5010, 11.2053).

**Table 3:** Segmentation performance of selected ED and ES frames of 1050 patients

Contour model	Dice (mean ± SD)	IoU (mean ± SD)	Hausdorff (mean ± SD)	Average Hausdorff (mean ± SD)	Successful frame count (total 2100)
EchoNet-Dynamic	0.8307 ± 0.0799	0.7172 ± 0.1011	37.5010 ± 17.8117	11.2053 ± 6.6333	2068
DTHR-SegStrain	0.9073 ± 0.0523	0.8342 ± 0.0800	17.1316 ± 7.4844	7.1883 ± 3.9248	2100
Unity-GLS	0.9097 ± 0.0774	0.8410 ± 0.0969	19.3002 ± 26.0130	7.5315 ± 6.0076	2085

### Hybridization of Segmentation Model and Strain Calculation Algorithm

**Fig. 3:**
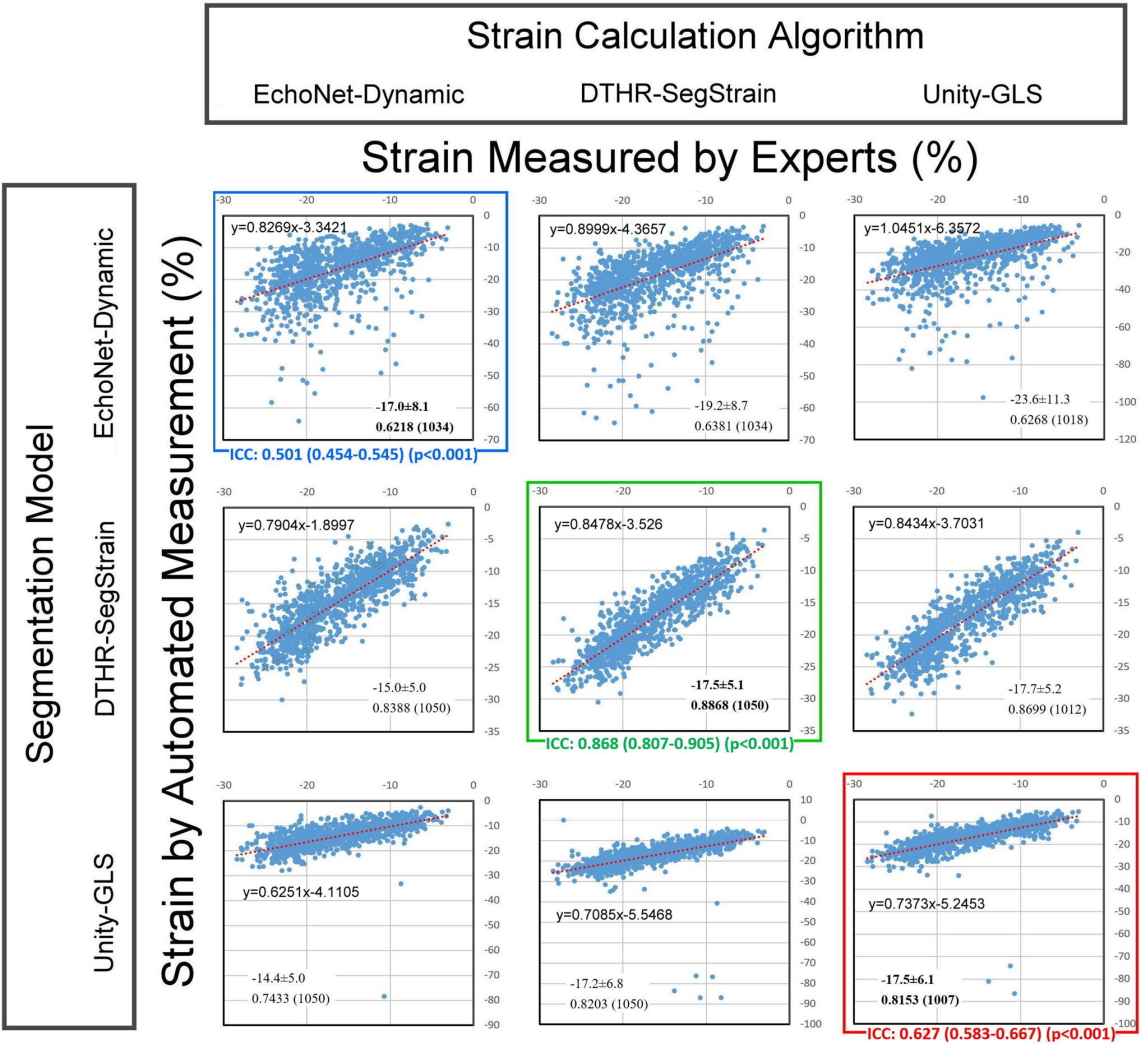
Scatterplots of LVGLS from 1050 individuals in a hybridization experiment. Proprietary combinations are identified with blue square for EchoNet-Dynamic, green square for DTHR-SegStrain, and red square for Unity-GLS. The averaged strain values are provided as mean ± SD; Spearman’s *ρ* is reported to the fourth decimal place. The numbers in parentheses indicate the number of videos in the validation dataset (out of 1050) for which GLS could be successfully calculated by each hybridization combination

Three segmentation models (DTHR-SegStrain, Unity-GLS, and EchoNet-Dynamic) were combined with three strain calculation algorithms (DTHR-SegStrain, Unity-GLS, EchoNet-dynamic) to yield 9 scatterplots against the expert-derived strain values (Fig. 3).

### Image Quality and Model Performance

View classification confidence has been reported as a surrogate marker for image quality [17]; we computed the classification confidence scores for all 1050 A4C views in the validation cohort. We then calculated the relative difference between the DTHR-SegStrain results and expert-derived strain values using the formula:

$$\left(DTHR-SegStrain-ExpertStrain\right)/ExpertStrain$$

Spearman’s rank correlation analysis revealed a statistically significant weak negative correlation (*ρ* =  − 0.2112, *p* < 0.0001), indicating that higher image quality was associated with a smaller relative difference between the model-derived and expert strain measurements.

### Scatterplots and Linear Regression Between AI-Strain and Expert Strain Values

Figure 3 summarizes Spearman’s *r* value of nine hybridized strain methods comparing to expert strain values. DTHR + DTHR had the highest correlation with expert strain. The ICC between three proprietary combinations and expert strain values were DTHR + DTHR: 0.868 (0.807–0.905), *p* < 0.001; Unity + Unity: 0.627 (0.583–0.667), *p* < 0.001; and EchoNet + EchoNet: 0.501 (0.454–0.545), *p* < 0.001.

Compared with proprietary combination, the EchoNet-dynamic contour will cause a higher absolute strain value (EchoNet + DTHR vs. DTHR + DTHR *n* = 1034: − 19.18 ± 9.72 vs. − 17.51 ± 5.13, *p* < 0.001; EchoNet + Unity vs. Unity + Unity n = 979: − 23.52 ± 11.12 vs. − 17.31 ± 8.86, *p* < 0.001). Interestingly, the EchoNet-dynamic strain algorithm will cause a lower absolute strain value (DTHR + DTHR vs. DTHR + EchoNet *n* = 1050: − 17.53 ± 5.11 vs. − 14.95 ± 5.04, *p* < 0.001; Unity + Unity vs. Unity + EchoNet *n* = 1007: − 17.46 ± 6.14 vs. − 14.43 ± 5.04, *p* < 0.001). On the other hand, either the change of segmentation contour (DTHRUnity) or the strain calculation algorithm (DTHR Unity) did not cause remarkable strain value deviation (DTHR + DTHR vs. Unity + DTHR *n* = 1050: − 17.52 ± 5.11 vs. − 17.24 ± 6.83, *p* = 0.1151; DTHR + DTHR vs. DTHR + Unity *n* = 1012: − 17.585.10 vs. − 17.665.19, *p* = 0.0607).

### Bland Altman Analysis Between AI-Strain and Expert Strain Values

**Fig. 4:**
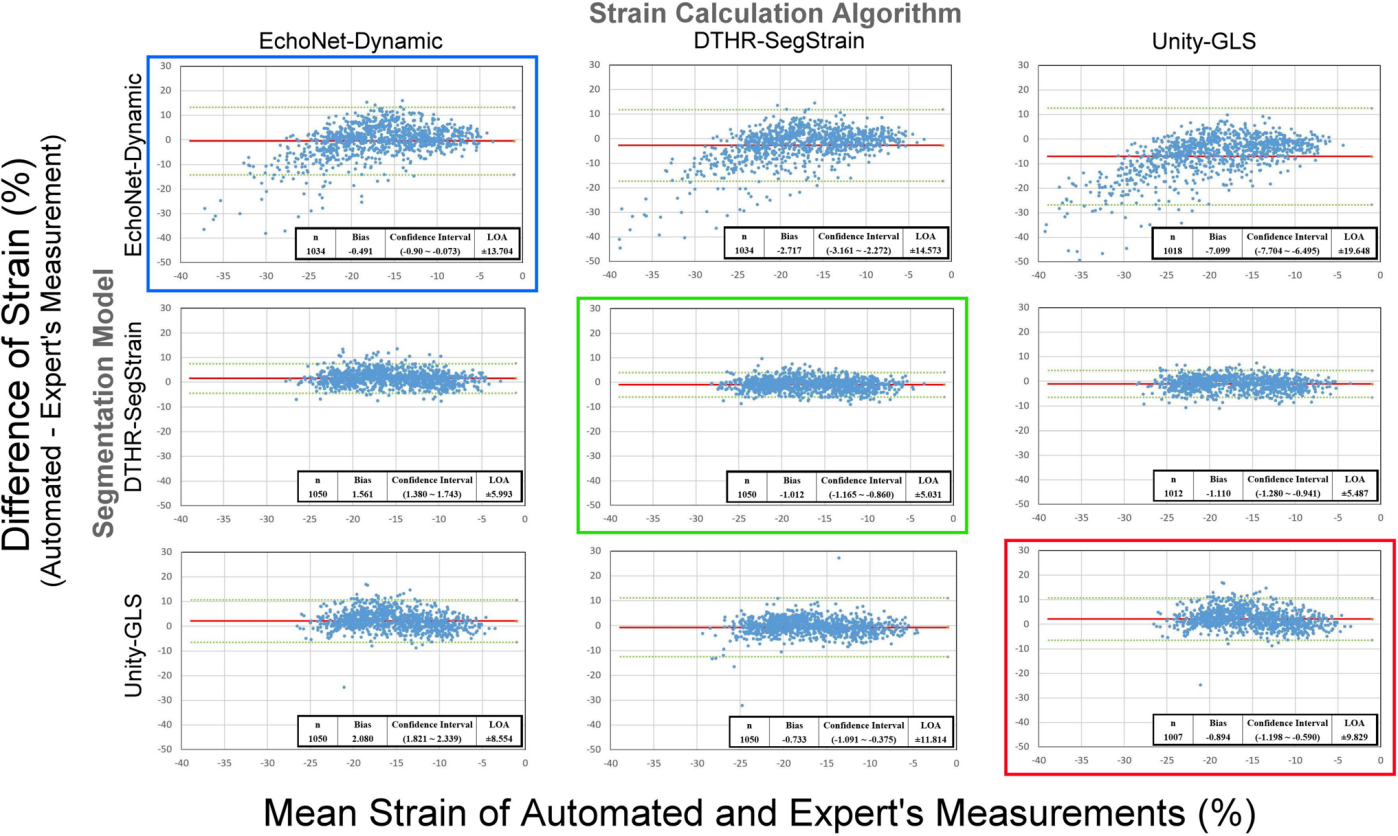
Bland Altman analysis between AI-strain and expert strain values. Proprietary combinations are identified with a blue square for EchoNet-Dynamic, a green square for DTHR-SegStrain, and a red square for Unity-GLS. LOA: limits of agreement; n: the numbers in parentheses indicate the number of videos in the validation dataset (out of 1,050) for which GLS could be successfully calculated by each hybridization combination

Figure 4 summarizes the results of Bland–Altman analysis. All three proprietary combinations had small mean differences compared to expert strain, but DTHR + DTHR had the lowest limits of agreement (LOA) (bias, LOA: DTHR + DTHR − 1.012, ± 5.031; Unity + Unity − 0.894, ± 9.829; EchoNet + EchoNet: − 0.491, ± 13.704).

The DTHR-SegStrain contour maintains good agreement regardless of the strain calculation algorithm used (bias, LOA: DTHR + EchoNet 1.561, ± 5.993; DTHR + Unity − 1.110, ± 5.487). However, the agreement of EchoNet-dynamic and Unity-GLS decreases when using other strain algorithms (bias, LOA: EchoNet + DTHR − 2.717, ± 14.573; EchoNet + Unity − 7.099, ± 19.648; Unity + DTHR − 0.733, 11.814; Unity + EchoNet 2.080, ± 8.554).

### ROC Analysis to Predict the LVGLS Value Above or Below the Normal Range

**Fig. 5:**
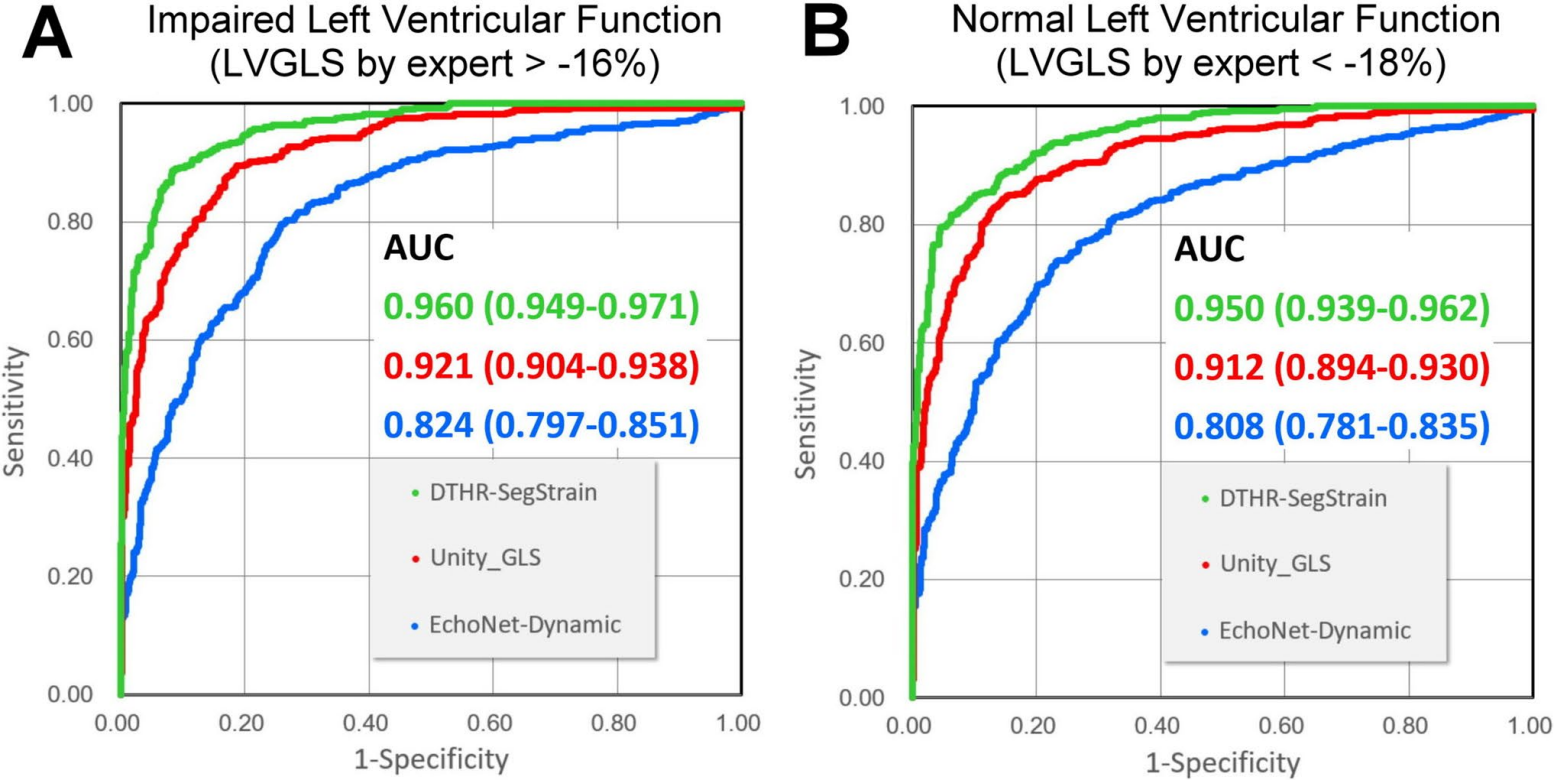
ROC analysis to predict the LV function by AI-strain with the proprietary combination. Among the 1050 validation videos, expert strain >  − 16% was defined as impaired LV function **A** and expert strain <  − 18% as normal LV function **B**. ROC curves were used to evaluate the discriminative performance of the three proprietary combinations. AUC, area under the curve; LVGLS, left ventricular global longitudinal strain

Clinically, a LVGLS value of less than − 18% is considered normal, while a value greater than − 16% is indicative of impaired LV function [20]. ROC analysis was performed to evaluate the diagnostic performance of these three proprietary combinations of AI-based strain methods. To predict impaired LV function, the AUC values were 0.960 (0.949–0.971) for DTHR + DTHR, 0.921 (0.904–0.938) for Unity + Unity, and 0.824 (0.797–0.851) for EchoNet + EchoNet (Fig. 5A). To predict normal LV function, the AUC values were 0.950 (0.939–0.962) for DTHR + DTHR, 0.912 (0.894–0.930) for Unity + Unity, and 0.808 (0.781–0.835) for EchoNet + EchoNet (Fig. 5B).

### Application of DTHR-SegStrain to Apical 2-Chamber, 3-Chamber, and Averaged LVGLS

We evaluated the agreement between DTHR-SegStrain-derived LVGLS and expert reference values across apical 2-chamber (A2C), apical 3-chamber (A3C), and the averaged 3-view LVGLS (Table S1, Fig. S1, S2). In the A2C view, DTHR-SegStrain achieved Spearman’s *ρ* of 0.8450 and an ICC of 0.833 (0.814–0.851), with a bias of 0.488 and LOA of ± 5.61. For the A3C view, the model yielded a ρ of 0.8585 and an ICC of 0.853 (0.836–0.896), with a slightly higher bias of 1.143 and LOA of ± 5.11. When averaging GLS across A4C, A2C, and A3C views, the model achieved the strongest correlation with expert values (*ρ* = 0.9193, ICC = 0.913 (0.903–0.923), and the narrowest LOA (± 3.51%), with a minimal bias of 0.21%.

## Discussion

We demonstrated that the DTHR-SegStrain provided accurate semantic segmentation with high temporal consistency. This improvement eliminates the need for a smoothing filter in AI-assisted strain analysis. In a hybridization experiment, we compared our model with two other AI-assisted strain calculation methods. The results showed that segmentation and strain computation can be decoupled. Importantly, we validated this feasibility not only in A4C-derived LVGLS, but also in A2C, A3C, and averaged LVGLS measurements. These findings support the idea that a well-designed segmentation model can reduce the variability introduced by proprietary strain algorithms. This, in turn, enhances the reliability and validity of LVGLS measurements in clinical practice.

### Advantages of V-SwinT and Regression-Based Design for Strain Calculation

Our framework uses a V-SwinT backbone combined with a regression head and an FCN-style multiscale feature fusion. This design improves both computational efficiency and accuracy, addressing the limitations seen in prior methods such as EchoNet-Dynamic and Unity-GLS.

EchoNet-dynamic relies on a DeepLab V3 + architecture that includes a CNN backbone, neck, and segmentation head. While this approach excels in extracting comprehensive segmentation maps for cardiac contours, it is computationally intensive and not optimized for direct strain calculation. Similarly, Unity-GLS uses HRNet architecture with a heatmap head for feature point localization. Although robust in many settings, heatmap predictions can occasionally fail, leading to positional errors (Figs. 7A and 8C, D).

In contrast, our use of a regression head avoids post-processing steps, eliminates heatmap quantization errors, and removes the need for manually designed ground truth heatmaps [21]. Previous CNN-based regression approaches struggled with stability and precision, but V-SwinT overcomes these issues. Our DTHR-SegStrain not only reduces computational overhead but also effectively captures the cardiac shape and spatiotemporal relationships. These improvements restore the feasibility of regression heads in LVGLS calculation.

In addition, the FCN-style multiscale fusion in our architecture balances global context with fine local details. Unlike EchoNet-Dynamic’s segmentation head, our regression head generates compact, parameterized outputs with minimal distortion. This makes our approach more efficient and more applicable in clinical settings.

### Importance of Spatiotemporal Consistency on Echocardiography

CNN-based neural networks have achieved significant success in static image semantic segmentation. This success naturally led to the use of 3D spatiotemporal convolutions for dynamic cardiac ultrasound. While this approach performs well in estimating LVEF in high-quality, preselected echocardiographic images, it faces limitations when applied to LVGLS computation.

**Fig. 6:**
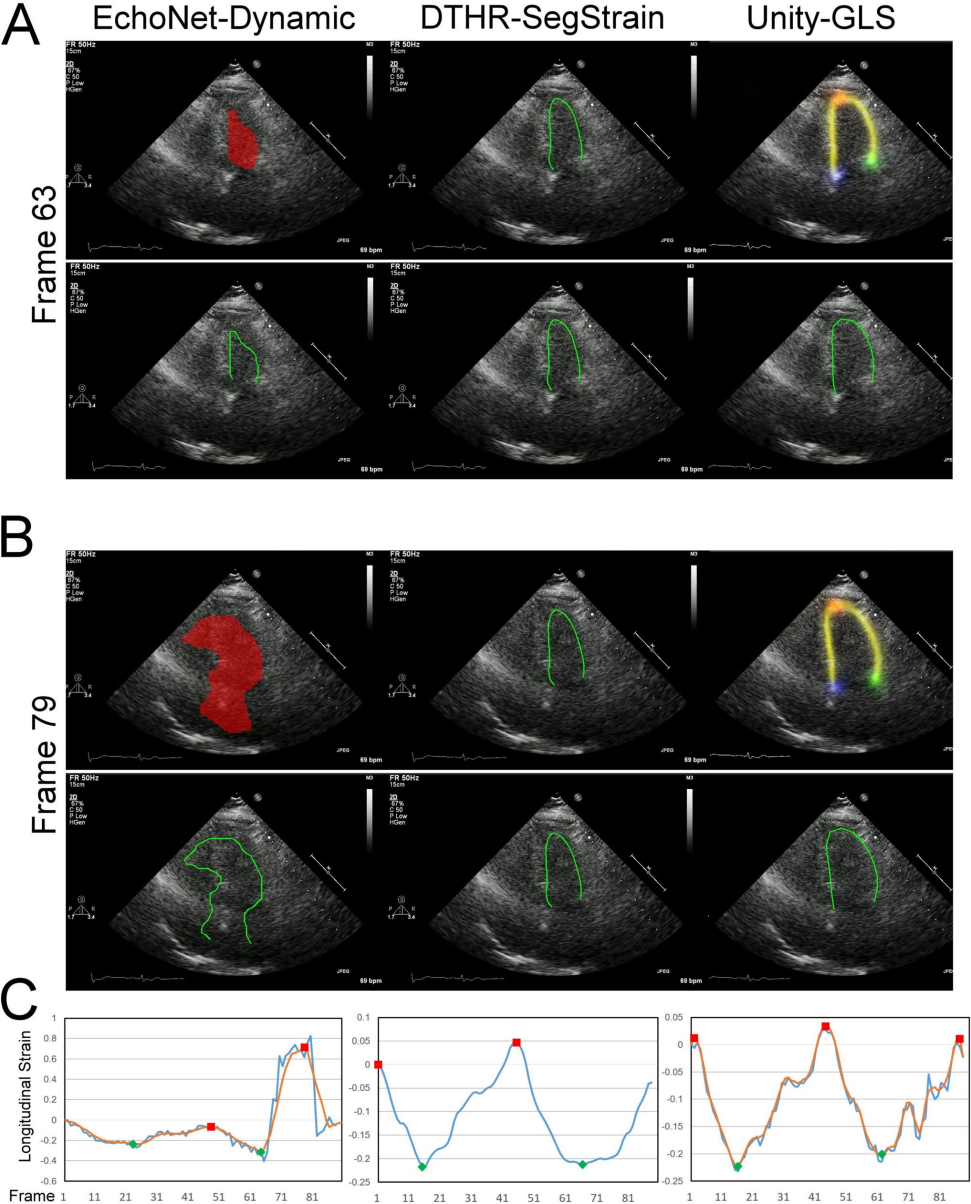
Performance of three AI models under poor image quality. Poor image quality results in irregular and temporally inconsistent LV segmentation masks in EchoNet-Dynamic (left panels), leading to highly jittery strain-to-time curves and errors in identifying ED and ES time points (C, bottom left). Although Unity-GLS successfully computes LVGLS, the strain-to-time curves exhibit significant jittering (C, bottom left, blue curve) that cannot be resolved even with smoothing filters (C, bottom left, orange curve). Please refer to video S2 for dynamic visualization

**Fig. 7:**
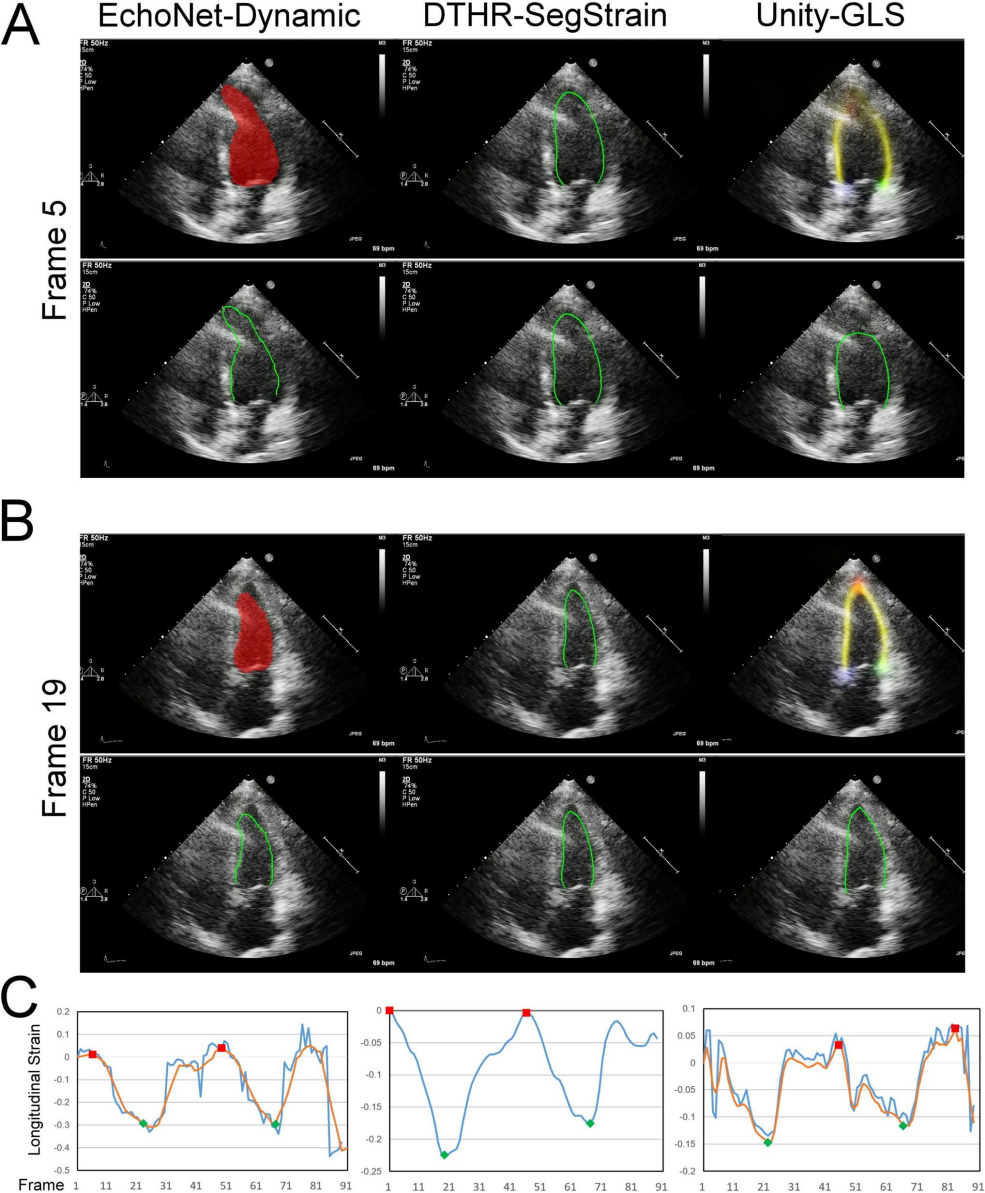
Performance of three AI models under reverberation artifacts. Reverberation artifacts cause distorted LV mask in EchoNet-Dynamic (**A, B** upper-left panel, red mask) and truncated apex in Unity-GLS (**A** upper-right panel, faded-out red shadow and yellow stripe at LV apex). These errors further affect the accuracy of myocardial length estimation, leading to erroneous strain-to-time curves (**C** left and right blue curves) that cannot be corrected by smoothing filters (**C** orange curves). Please refer to video S3 for dynamic visualization

**Fig. 8:**
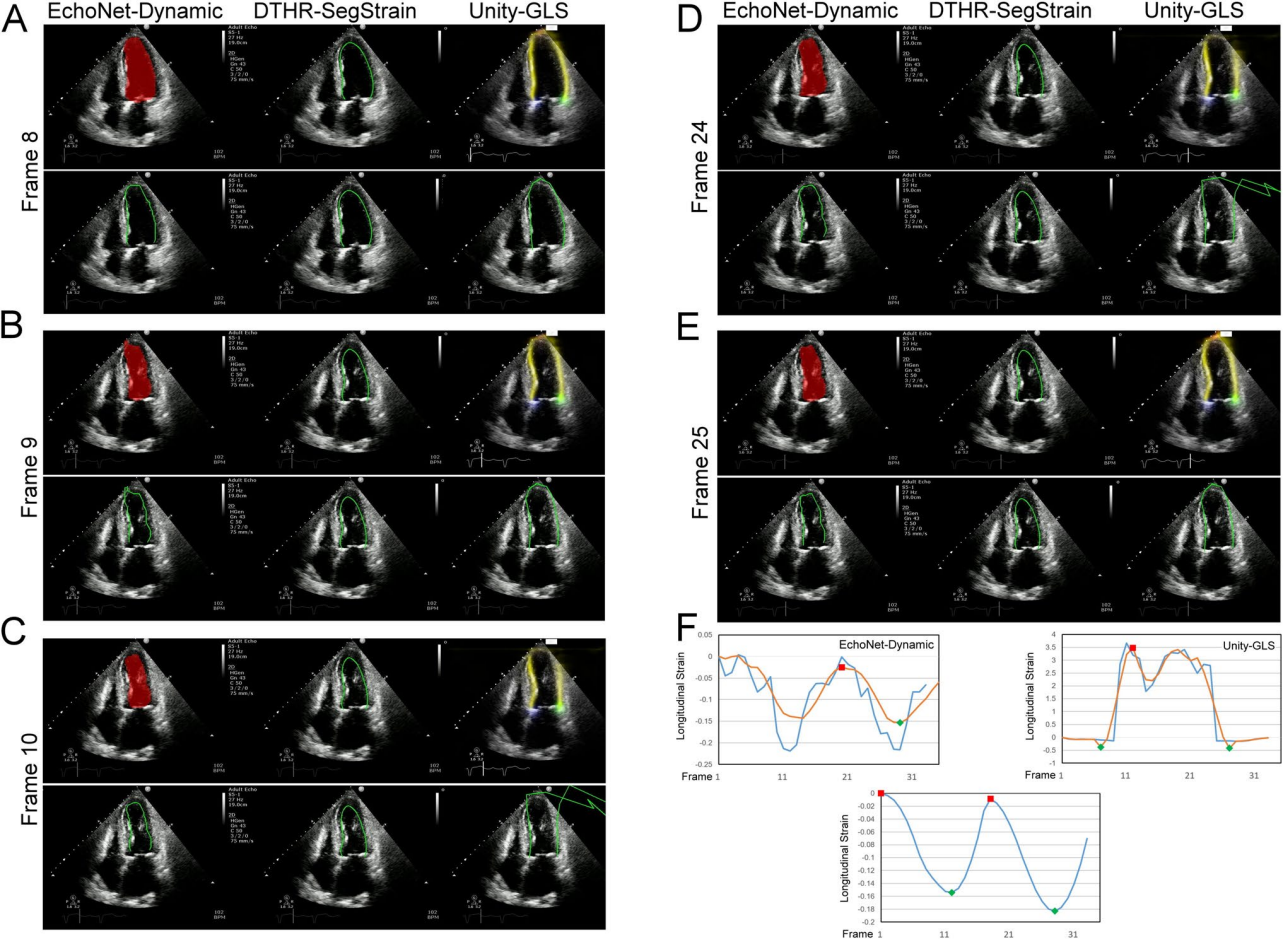
Performance of three AI models under poor delineation of LV apex. A-E, selected frames to demonstrate the segmentation performance of AI models. The Unity-GLS struggles with the low confidence of its LV border heatmap, resulting in noisy contour lines during LV border calculations (**C, D** right panels, yellow stripes and green contours). The temporal consistency of EchoNet-Dynamic deteriorates further in poor apical delineation (**A–E** left panels, red masks and corresponding green contours), leading to a reduced number of ED/ES points identified by the strain algorithm (**F**). Both EchoNet-Dynamic and Unity-GLS failed identification of the end-diastolic and end-systolic point of the strain-to-time curve (**F** Errors exist in the number and placement of red (end-diastolic) and green (end-systolic) points on the left and right strain-curve panels). In this image, the blood–muscle border at the apical lateral wall is more easily identifiable in the video than in the static frame. And the spatiotemporal consistency of DTHR-SegStrain effectively addresses this issue (**A–F** middle panels). Please refer to video S4 for dynamic visualization

**Fig. 9:**
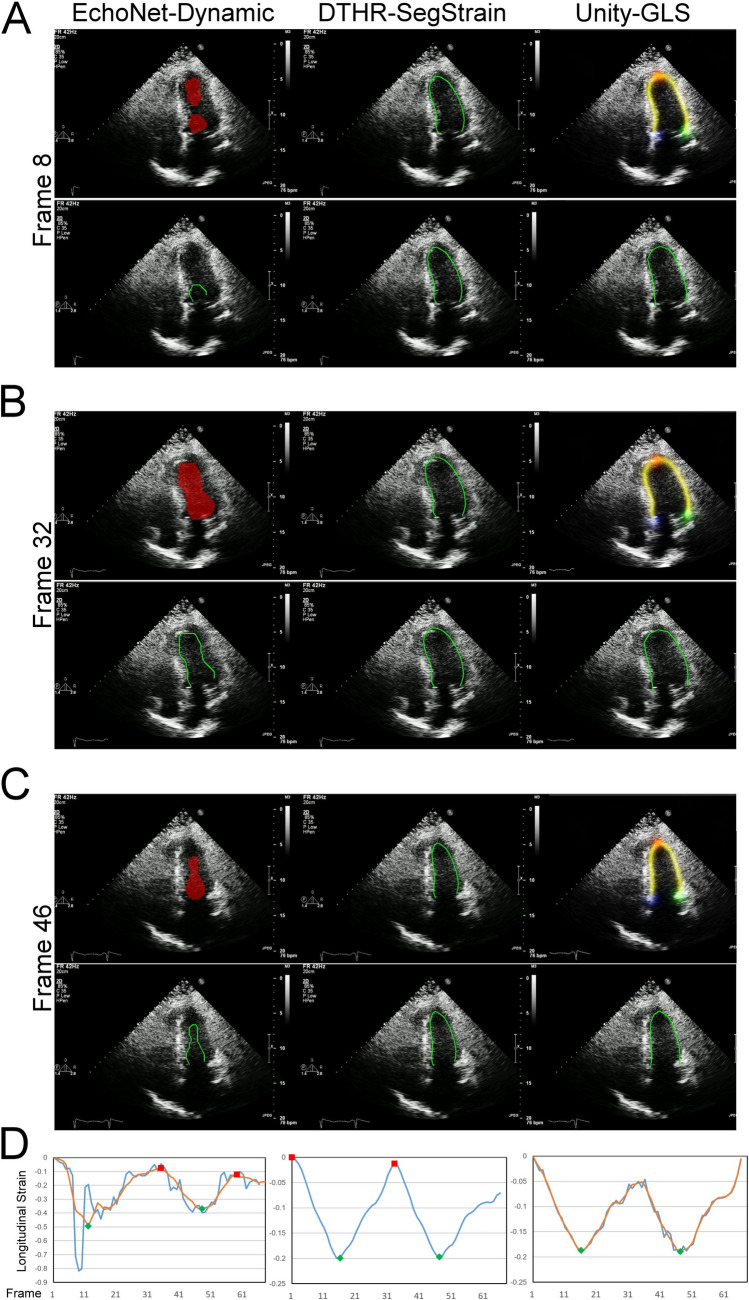
Irrational end-diastolic or end-systolic time points due to a lack of temporal consistency. Due to the end-diastolic/end-systolic threshold limitations in the Unity-GLS strain algorithm, no end-diastolic point was identified for this video (D, right curves, missing red dots), making LVGLS calculation impossible. Additionally, fragmented masks were observed during segmentation with EchoNet-Dynamic, resulting in a fragmented, irregular LV mask (A-C, upper-left panel) and an anatomically inconsistent contour (A&C, bottom left panel). Please refer to video S5 for dynamic visualization

Unlike traditional object segmentation, cardiac chambers are not distinct objects with clear boundaries. Instead, they are separated by thin, fast-moving valves or structures like the atrial-ventricular septum, which has inconsistent echogenicity. When each frame is segmented independently, the resulting pixel masks tend to have irregular, jagged borders and poor temporal coherence (Figs. 6, 7, 8, and 9, red masks). This lack of temporal consistency causes instability in the strain-to-time curve. Researchers often apply smoothing filters (Figs. 2, 6, 7, 8, and 9 orange curves) and rely on precise criteria for identifying ED and ES timepoints to reduce such fluctuations.

The performance of CNN-based segmentation is further limited by several operator-related factors, including foreshortened views, suboptimal acquisition angles, patient-related factors, including translational motion and occasional out-of-plane motion, and ultrasound-specific artifacts such as reverberation, refraction, side lobes, mirror artifacts, and near-field clutter [22]. These challenges contribute to increased segmentation failure rates, as shown in Figs. 6, 7, 8, and 9 and supplementary videos. For example, EchoNet-Dynamic often produces uneven contours (Fig. 6) and fragmented segmentation masks (Fig. 9), sometimes even including masks for other cardiac chambers or regions outside the heart [14]. Unity-GLS also fails when the confidence of its LV border heatmap is low (Fig. 7), resulting in noisy contours (Fig. 8). These inaccuracies lead to excessive contour lengths that significantly distort strain values. To compensate, Unity-GLS applies length-based thresholds at ED and ES in its strain algorithm. However, this approach can sometimes abandon strain calculations (Fig. 9). This explains why Unity-GLS did not achieve a 100% contour-to-strain conversion rate in our experiments.

Transformer-based architectures leverage self-attention to effectively capture long-range dependencies, making them particularly suitable for echocardiographic image segmentation where cross-regional relationships and boundary ambiguities are common. Compared with convolutional neural networks, they provide stronger global contextual modeling, enhanced robustness to artifacts, and better integration of local and global features. Prior transformer-based approaches such as SegFormer and SwinT [14] have demonstrated benefits in echocardiographic segmentation, but they are originally designed for static 2D frames, thus overlooking temporal dynamics. V-SwinT addresses this limitation by extending SwinT’s shifted window mechanism into three dimensions, incorporating the temporal axis. In this design, global self-attention enforces spatial consistency within each frame, while 3D shifted windows introduce temporal consistency across consecutive frames. Together, these mechanisms enable V-SwinT to achieve robust spatiotemporal consistency, making it particularly well suited for echocardiographic video segmentation tasks. Our present study shows that V-SwinT addresses these challenges with its superior spatiotemporal consistency. Consequently, the strain-to-time curves require no additional smoothing filters, simplifying strain algorithm design and reducing measurement variability.

### More Segmentation, Less Calculation: Insights from the Hybridization Experiment

Our study pioneers the concept of separating segmentation contour generation from strain calculation algorithms. In our experiments, the EchoNet-Dynamic segmentation often produces exaggerated GLS absolute values. These values are subsequently corrected by its smoothing-based algorithm. While this approach achieves acceptable mean differences, the LOA exceed clinically tolerable thresholds. This compromises its sensitivity in detecting subtle myocardial dysfunction. By analyzing strain-to-time curves, we found that static segmentation, which lacks temporal consistency, relies heavily on algorithmic adjustments. EchoNet-Dynamic achieves speckle-tracking regularization effects through smoothing, while Unity-GLS calculates strain by averaging ED and ES lengths. However, these fixes introduce additional GLS variability, ultimately limiting their clinical reliability.

Conversely, our results demonstrate that segmentation contours with robust spatiotemporal consistency can produce reliable GLS results, even when paired with third-party algorithms. In contrast, the contours from DTHR-SegStrain and Unity-GLS lose validity when processed with EchoNet-Dynamic’s strain algorithm, suggesting that EchoNet-Dynamic’s algorithm is tailored to correct biases in its own segmentation outputs. This dependency underscores the limitations of strain calculation algorithms designed to compensate for segmentation errors, as they may lack generalizability. Consequently, we propose leveraging the high-quality and temporally consistent segmentation to simplify strain calculation algorithms. By reducing reliance on algorithmic corrections, this approach democratizes the strain estimation process, minimizing variability and improving reliability.

### Pave the Way for Regional Strain Analysis

Regional strain offers valuable insights into both left ventricular RWMA and asynchronous contraction. However, its previous application has been largely limited by two major challenges. First, to compute regional strain, each apical scanning view (A4C, A2C, and A3C) must be divided into six segments. The five division points between septal and lateral annuli are inherently less stable on a frame-by-frame basis. In the presence of RWMA, the initial six equal divisions at end-diastole often correspond to uneven segment lengths at end-systole. Similarly, in asynchronous contraction, the time lag between septal and lateral walls and possible reverse LV septal contour might introduce discrepancies that current modeling-based software would struggle to capture accurately. Although prior studies have attempted to address this limitation using dynamic time warping to improve AI-based segmentation of regional longitudinal strain [8], such approaches still require higher spatiotemporal resolution. Our V-SwinT-based LV contour segmentation provides endocardial contours with high spatiotemporal resolution. Furthermore, through its regression head, it outputs explicit coordinate points along the contour, enabling reconstruction of continuous curves suitable for dynamic time warping. This provides a stronger methodological foundation for future studies of regional longitudinal strain.

### Study Limitations

This study has several limitations. Our experiments focused exclusively on longitudinal strain, without including circumferential or regional strain measurements. Although global circumferential strain is clinically relevant, its estimation in routine echocardiography is limited by technical challenges such as out-of-plane motion and oblique slicing in short-axis imaging. This hinders the availability of reliable expert strain measurements for validation. For regional strain, previous work demonstrated that it can be estimated using dynamic time warping when accurate contours are available [8]. However, both EchoNet-Dynamic and Unity-GLS lack regional strain results for comparison. Besides, our current combination of segmentation model and strain algorithm might not be optimal. Although the model succeeded in all the strain calculation tasks in the 1050-video, this likely reflects a selection bias of our validation dataset. Studies with extremely poor image quality, severe artifacts, or arrhythmias were naturally excluded from validation because they also could not yield expert-strain values. Such a limitation is common to all commercially available software.

Furthermore, our study primarily utilized high-end echocardiography machines. While we developed a dynamic programming-based approach to identify ED and ES timepoints without relying on ECG signals, this method has not yet been tested on hand-held ultrasound devices. Additionally, most phenotypes in open-source datasets exhibited normal ventricular morphology. Although we supplemented these with institutional training data, atypical morphologies, such as spade-shaped left ventricles or reversed interventricular septal contours, remained underrepresented. To mitigate such epistemic uncertainty [23] and improve robustness, future work could leverage domain-specific pre-training using large-scale cardiac CT or CMR datasets to provide anatomical priors for echocardiography. Echocardiography-specific challenges, including probe instability, respiratory-induced cardiac motion, and lung-induced signal occlusion, could be addressed through architectural fine-tuning on artifact-rich echocardiographic images, for example using shape-constrained networks or multi-scale attention mechanisms to deal with aleatory uncertainty [23]. Finally, further studies are needed to externally validate the model and ensure its generalizability across diverse clinical scenarios.

## Conclusion

The DTHR-SegStrain leverages a video transformer architecture for spatiotemporal feature extraction, enabling the generation of temporally consistent LV contours via the regression head. This approach eliminates the need for post-processing steps, such as converting segmentation mask or heatmap into contours, and avoids reliance on additional smoothing filters. By allowing direct myocardial length measurement, this model minimizes variability and simplifies LVGLS computation, improving its reliability and clinical applicability.

## Supplementary Information

Below is the link to the electronic supplementary material.

ESM 1 (MP4 794 KB)

ESM 2 (MP4 0.99 MB)

ESM 3 (MP4 0.99 MB)

ESM 4 (MP4 417 KB)

ESM 5 (MP4 733 KB)

ESM 6 (PDF 1.76 MB)

## Data Availability

De-identified patient echocardiographic data reported in this study cannot be deposited in a public repository because of regulations from the research ethics committee and prohibition from the hospital administration. Processed datasets that support the findings of this study will be shared on reasonable request to the corresponding author.
