# Supplementary material for: Video Transformer for Segmentation of Echocardiography Images in Myocardial Strain Measurement
Source: J Imaging Inform Med. 2025 Sep 17;39(3):2661–79. doi: 10.1007/s10278-025-01682-5 (PMC13230454; doi:10.1007/s10278-025-01682-5)
Supplement: Supplementary file 6 — (PDF 1.76 MB) [file 10278_2025_1682_MOESM6_ESM.pdf]

SUPPLEMENTARY MATERIAL

Title: Video Transformer for Segmentation of Echocardiography Images in Myocardial Strain Measurement

Journal name: Journal of Imaging Informatics in Medicine

Kuan-Chih Huang[1,2] • Chang-En Lin, MD[3] • Donna Shu-Han Lin[4] • Ting-Tse Lin[3] • Cho-Kai Wu[3] • Geng-Shi Jeng[5] • Lian-Yu Lin[3] • Lung-Chun Lin[3]

From the

1. Graduate Institute of Clinical Medicine, College of Medicine, National Taiwan University, Taipei, Taiwan
2. National Taiwan University Hospital, Hsin-Chu Branch, Hsinchu, Taiwan, Taiwan
3. Section of Cardiology, Department of Internal Medicine, National Taiwan University Hospital, Taipei, Taiwan
4. Division of Cardiology, Department of Internal Medicine, Shin Kong Wu Ho-Su Memorial Hospital, Taipei, Taiwan
5. Institute of Electronics, National Yang Ming Chiao Tung University, Hsinchu, Taiwan

Corresponding author: Lung-Chun Lin,

E-mail: anniejou@ms28.hinet.net

Contents:

§ Experiment S1-3 model development and fine-tune

§ Dynamic-Programming ED–ES (DP-EDES) Algorithm

§ Application of DTHR-SegStrain to A2C, A3C and averaged GLS

§ Video S1 corresponds to Figure 2

§ Video S2 corresponds to Figure 6

§ Video S3 corresponds to Figure 7

§ Video S4 corresponds to Figure 8

§ Video S5 corresponds to Figure 9

Experiment S1: Frame-by-Frame LV Endocardial Contour Regression with Video Swin Transformer

In this experiment, we repurpose the Video Swin Transformer, initially designed for video-level classification, to perform single-frame coordinate regression of the left ventricular (LV) endocardial contour. By feeding the model a short spatiotemporal clip surrounding each target frame (13 frames before + target frame + 14 frames after), we evaluate whether V-SwinT can leverage temporal context to accurately predict the 49 (x, y) contour points of the center target frame.

LV Endocardial Contour Training Set

The preliminary experiments (S1–S3) were all trained using the datasets listed below. As these experiments were conducted during the early phase of the study, the datasets were eventually incorporated into the 1,282-institutional dataset described in the main text. This training set comprises manually annotated left-ventricular endocardial contours.

- **Annotation and Data Source**

(institutional dataset: IQ, normal ventricular function; D, very poor acquisitions; DCMP, dilated cardiomyopathy; RWMA, with regional wall motion abnormality)

  - From the previous IDOL training set (IQ set 1 + FISU), annotations on frames of a complete cardiac cycle.
  - Additional batch 2 (D + IQ set 2), annotations on end-diastolic to end-systolic frames.
  - Additional batch 3 (DCMP+RWMA), annotations on end-diastolic to end-systolic frames.

- **Dataset Split**
  - **Training:** 1,097 videos (~23,000 frames)
  - **Validation:** 109 videos (~2,300 frames)

- **Preprocessing for V-SwinT input**
  - Original frame size: 600 × 800 pixels
  - Center-padding and resizing to 168 × 168 pixels, or 192 × 192 pixels
  - Temporal length:
    - For each target frame, extract a clip of 28 consecutive frames: 13 preceding + target + 14 following
    - If fewer than 28 frames are available at the start or end of a video, pad with the nearest edge frame to reach 28 frames

- **Data Augmentation**
  - Brightness adjustment: random additive offsets with a variance of 75
  - Contrast adjustment: random multiplicative factor up to 0.3
  - Geometric augmentations
    - Translation: random shift of up to ±6.25 % of the image width/height

        - Scaling(zoom): random zoom in/out by up to ±20 %t
        - Rotation: random rotations within ±40°

- **Contour output structure**
    - 49 ordered (x, y) points per frame delineating the LV endocardial boundary

Test Set

We evaluate all methods on another 320 ultrasound clips of the same institution, each annotated manually between end-diastole (ED) and end-systole (ES) over one cardiac cycle. The following chart includes frame evaluation results.

Performance Metrics

- **Dice Coefficient**: Measures volumetric overlap between the predicted and ground-truth LV masks.
- **Hausdorff Distance**: Quantifies the maximum contour deviation between predicted and manual endocardial lines.

Compared Models

1. **DeepSono V0 (IDOL)**
    - The baseline "In Defense of Online Learning" (IDOL) architecture employed in our previous model first predicts the left-ventricular myocardium mask, from which the endocardial contour is then extracted.
2. **YOLOv8-Pose M (All Videos)**
    - Applies a YOLOv8-Pose M backbone to the full 320-video set. It processes each frame independently, directly outputting the coordinates of the points that delineate the LV endocardial contour.
3. **YOLOv8-Pose M (Filtered)**
    - Use automatic algorithm to excludes clips containing more than two frames with erroneous LV endocardial predictions, resulting in 280 videos.
4. **Video Swin Transformer (V-SwinT)**
    - Frame-by-frame coordinate regression: for each center frame, the model ingests a 28-frame clip (13 before + target + 13 after) and directly regresses its 49 contour points using a spatiotemporal windowed Transformer backbone (no multi-scale fusion)
5. **Video Swin Transformer + FPN**
    - Frame-by-frame coordinate regression with enhanced feature pyramid: extends V-SwinT by adding a two-level FPN head to improve localization of the 49 contour points for each target frame.

Hausdorff Results of Experiment S1

Table EXP1-1

Summary table for all nine models, including the core Hausdorff distance box-plot statistics, the calculated IQR, and extra columns that describe each model's key architectural or data-processing differences:

Model	Backbone	FPN	Input Size(HxW)	Augmentation	Loss	Min Out.	Min W.	Q1	Median	Q3	Max W.	Max Out.	IQR
**DeepSono V0**	IDOL	–	384×384	–	–	–	0	8.3	11	14.6	23.8	117.8	6.3
**YOLOv8-Pose M 640**	YOLOv8-Pose M	–	640×640	–	–	–	2.1	6.8	8.9	11.5	18.6	198.4	4.7
**YOLOv8-Pose M 640 280,** Filtered to 280 videos (≤2 bad frames)	YOLOv8-Pose M	–	640×640	–	–	–	2.1	6.7	8.6	11	17.6	153.1	4.3
**V-SwinT-S 168**	V-SwinT-S	–	168×168	No	MSE	–	7.9	12.6	14.4	16.8	23.1	44.9	4.2
**V-SwinT-S-FPN 168**	V-SwinT-S	2-level features (512 output channels)	168×168	No	MSE	–	8.1	12.1	13.7	15.9	21.6	47.1	3.8
**V-SwinT-S-FPN 192**	V-SwinT-S	2-level features (512 output channels)	192×192	No	MSE	–	8.5	12.5	14.2	16.7	23	40.3	4.2
**V-SwinT-S-FPN 168 Aug**	V-SwinT-S	2-level features (512 output channels)	168×168	Yes	MSE	–	8.1	12.7	14.5	16.9	23.1	43.9	4.2
**V-SwinT-S-FPN 168 Aug MSE FPN768**	V-SwinT-S	2-level (768 output channels)	168×168	Yes	MSE	–	8.6	12.4	14.2	16.5	22.5	123.4	4.1
**V-SwinT-S-FPN 168 Aug S-MSE FPN512**	V-SwinT-S	2-level (512 output channels)	168×168	Yes	Scale-weighted MSE	–	7.6	12.8	14.7	17.6	24.7	45.7	4.8

- **Min W./Max W.** = whisker values (most extreme non-outliers) **Min Out./Max Out.** = most extreme points beyond 1.5 × IQR **Q1 / Median / Q3** = 25th/50th/75th percentiles. **IQR** = Q3 – Q1

Figure EXP1-1

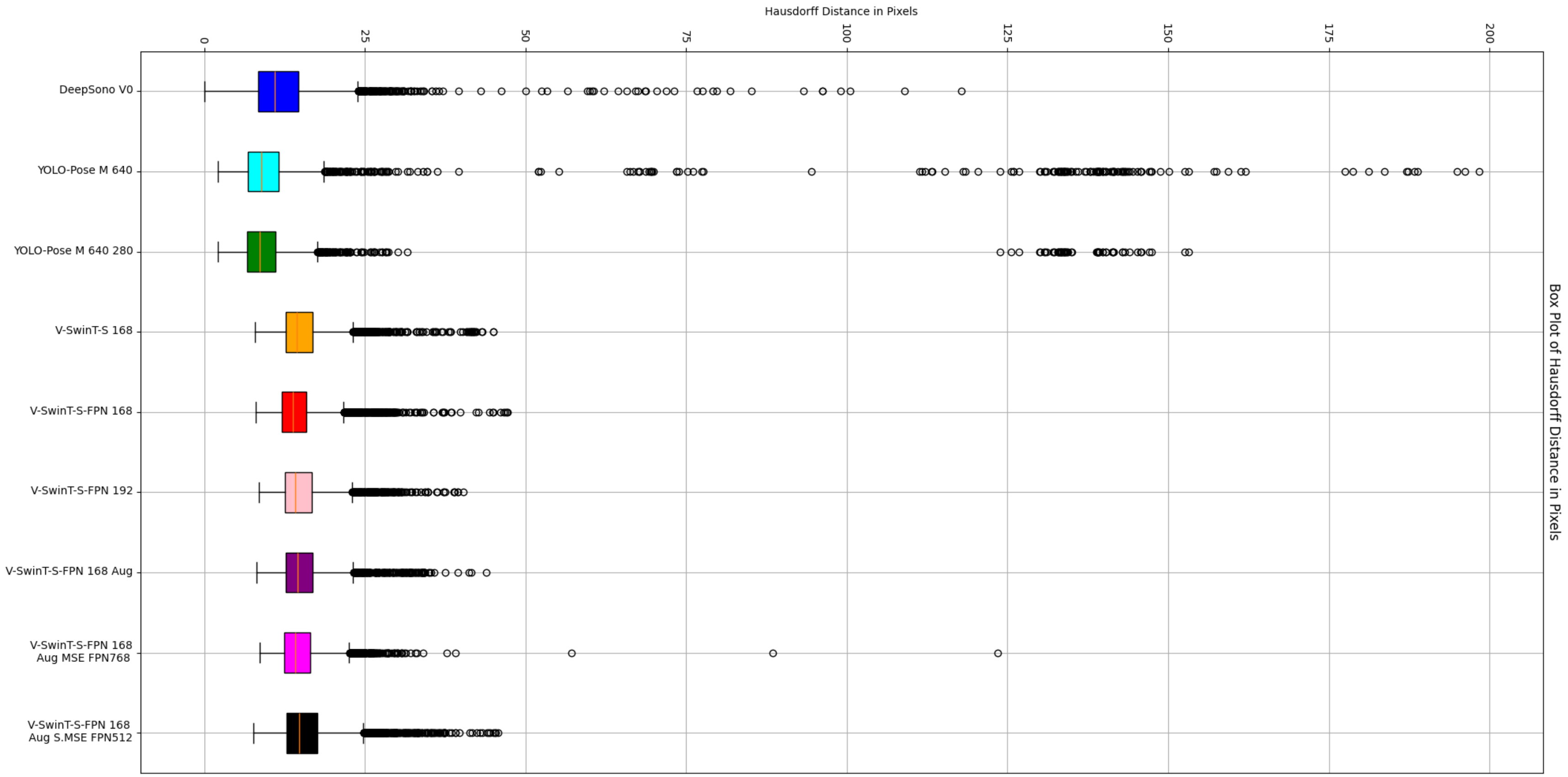

Dice Results of Experiment S1

Table EXP1-2

Model	Backbone	FPN	Input Size	Augmentation	Loss	Dice Min Out.	Dice Min W.	Q1	Median	Q3	Dice Max W.	Dice Max Out.	IQR
**DeepSono V0**	IDOL	–	384×384	–	–	0	0.839	0.902	0.926	0.945	0.993	–	0.043
**YOLOv8-Pose M 640**	YOLOv8-Pose M	–	640×640	–	–	0	0.885	0.931	0.949	0.962	0.988	–	0.031
**YOLOv8-Pose M 640 280,** Filtered to 280 videos (≤2 bad frames)	YOLOv8-Pose M	–	640×640	–	–	0	0.89	0.934	0.951	0.963	0.988	–	0.029
**V-SwinT-S 168**	V-SwinT-S	–	168×168	No	MSE	0.731	0.873	0.922	0.94	0.954	0.981	–	0.032
**V-SwinT-S-FPN 168**	V-SwinT-S	2-level features (512 output channels)	168×168	No	MSE	0.593	0.879	0.926	0.945	0.958	0.985	–	0.032
**V-SwinT-S-FPN 192**	V-SwinT-S	2-level features (512 output channels)	192×192	No	MSE	0.59	0.866	0.918	0.938	0.952	0.983	–	0.034
**V-SwinT-S-FPN 168 Aug**	V-SwinT-S	2-level features (512 output channels)	168×168	Yes	MSE	0.685	0.865	0.917	0.937	0.951	0.984	–	0.034
**V-SwinT-S-FPN 168 Aug MSE FPN768**	V-SwinT-S	2-level (768 output channels)	168×168	Yes	MSE	0	0.882	0.925	0.943	0.955	0.982	–	0.03
**V-SwinT-S-FPN 168 Aug S-MSE FPN512**	V-SwinT-S	2-level (512 output channels)	168×168	Yes	Scale-weighted MSE	0.547	0.856	0.912	0.934	0.949	0.982	–	0.037

Min W./Max W. = whisker values (most extreme non-outliers) Min Out./Max Out. = most extreme points beyond 1.5 × IQR Q1 / Median / Q3 = 25th/50th/75th percentiles. IQR = Q3 – Q1

Figure EXP1-2

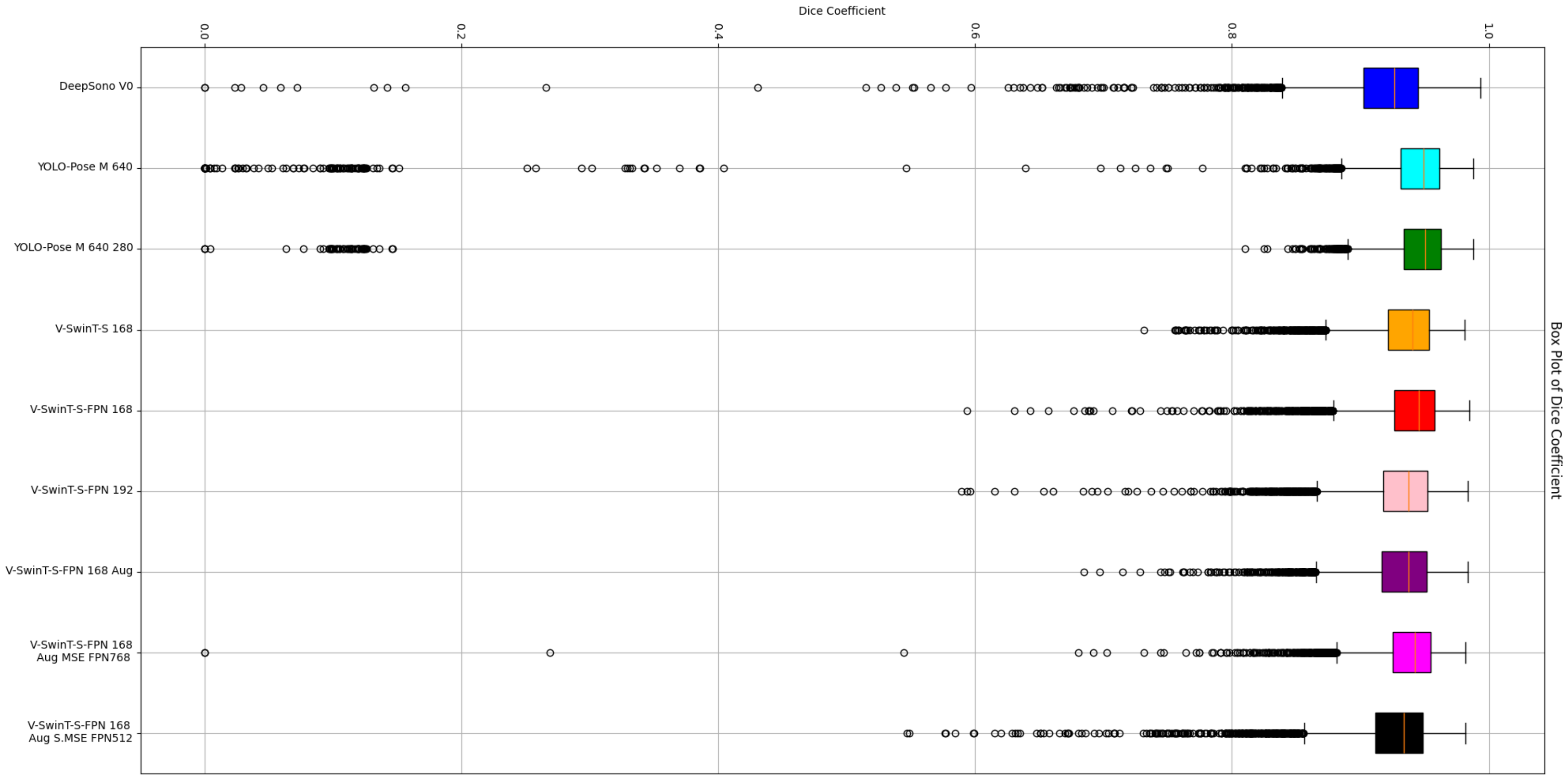

Model differences in a nutshell

- **DeepSono V0 (IDOL):** The baseline "In Defense of Online Learning" (IDOL) architecture employed in our previous model first predicts the left-ventricular myocardium mask, from which the endocardial contour is then extracted.
- **YOLO-Pose M:** The frame-based models employ a 640 × 640 YOLO-Pose network to predict the LV endocardial contour point coordinates on a per-frame basis. The "280" variant further excludes any video containing more than two frames with erroneous predictions, yielding a filtered set of 280 videos. Each frame is processed independently, with the network directly outputting the (x, y) coordinates that delineate the left-ventricular endocardial boundary.
- **V-SwinT-S** backbones leverage spatiotemporal Windowed MSA (Video Swin Transformer) to embed temporal context directly at the token level.
- **FPN** variants fuse two layers of multi-scale features before the regression head, improving robustness to scale.
- **FPN output dimensions**: two-level FPN that fuses multi-scale feature maps into a single feature map with **512 or 768 channels** (i.e., the output FPN feature map's channel dimension), which is then used to regress the 49 contour points of the target frame.
- **Input size** differences (168 vs. 192) affect spatial resolution.
- **Aug** (augmentation) introduces basic geometric transforms (scale/translation/rotation), brightness/contrast jitter to simulate variability,
- **Loss** variants: plain MSE treats all frames equally, whereas **S-MSE,** Scale-weighted MSE, to normalize the error weighting between contours with different sizes. Large contours can tolerate larger coordinate prediction error, while for smaller contours, smaller coordinate prediction error is required. However, the experiment result shows worse performance of the simple MSE loss.

Final Model Selection

Baseline Selection

We adopt the V-SwinT-S FPN 168 configuration as our baseline model for all subsequent V-SwinT variant comparisons, due to its low IQR,median and stable outlier performance, in dice and Hausdorff metrics

Loss Function Choice

Although the scale-weighted MSE (S-MSE) loss was designed to normalize errors across contours of different sizes, it yielded poorer Dice and Hausdorff metrics than plain MSE in our experiments. Consequently, we retain the simple MSE loss for all future model variants.

Effectiveness of Data Augmentation

Geometric (translation, scaling, rotation) and photometric (brightness, contrast) augmentations demonstrably improve robustness to variations in cardiac anatomy, probe angle, and patient positioning., see the following 2 figures:

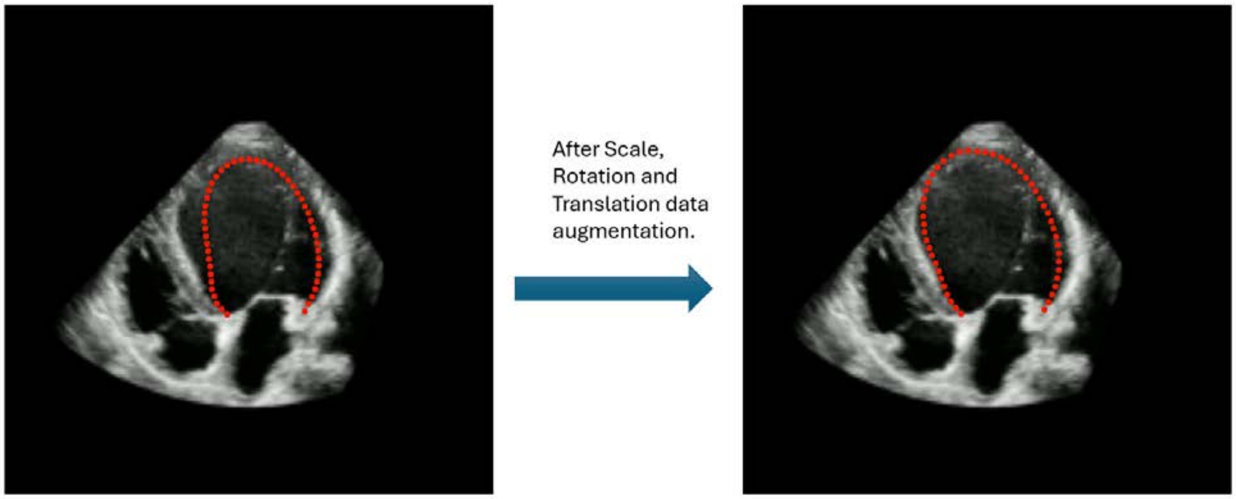

Experiment S2: V-SwinT variants

- **Annotation and Data Source & Data Split as Experiment S1**
- **Preprocessing for V-SwinT input**
  - Original frame size: 600 × 800 pixel
  - Center-padding and resizing to 224 × 224 pixel
  - Temporal length: Randomly cutting 64 consecutive frames， padding with the last frame if it is less than 64 frames
- **Data Augmentation**
  - Brightness adjustment: random additive offsets with a variance of 75
  - Contrast adjustment: random multiplicative factor up to 0.3
  - Geometric augmentations
    - Translation: random shift of up to ±6.25 % of the image width/height
    - Scaling(zoom): random zoom in/out by up to ±20 %
    - Rotation: random rotations within ±40°
- **Contour output structure**
  - 49 ordered (x, y) points per frame delineating the LV endocardial boundary

Compared Models

1. **DeepSono V0 (IDOL)**
   - The baseline "In Defense of Online Learning" (IDOL) architecture employed in our previous model first predicts the left-ventricular myocardium mask, from which the endocardial contour is then extracted.
2. **YOLOv8-Pose M (All Videos)**
   - Applies a YOLOv8-Pose M backbone to the full 320-video set. It processes each frame independently, directly outputting the coordinates of the points that delineate the LV endocardial contour.
3. **YOLOv8-Pose M (Filtered)**
   - Use improved automatic algorithm to exclude clips containing more than two frames with erroneous LV endocardial predictions, resulting in 280 videos.
4. **Full-Frame V-SwinT-B + FPN**

- Uses the larger Video Swin Transformer “B” backbone with a two-level FPN head. Ingests 64 consecutive frames at 224×224 pixel and outputs the full 64-frame sequence of endocardial contours.

5. **Single-Frame V-SwinT-S + FPN**
    - Employs the smaller “S” backbone with the same FPN head. Processes 28 frames at 168×168 pixel, but only predicts the central frame’s contour.

6. **Full-Frame V-SwinT-B + FPN**
    - Uses the smaller Video Swin Transformer “S” backbone with a two-level FPN head. Ingests 64 consecutive frames at 168×168 pixel and outputs the full 64-frame sequence of endocardial contours.

Hausdorff Results of Experiment S2

Table EXP2-1

Model	Backbone	FPN	Input Size(HxW)	Augmentation	Loss	Min Out.	Min W.	Q1	Median	Q3	Max W.	Max Out.	IQR
**DeepSono V0**	IDOL	–	384×384	–	–	–	0	8.3	11	14.6	23.8	117.8	6.3
**YOLOv8-Pose M 640**	YOLOv8-Pose M	–	640×640	–	–	–	2.1	6.8	8.9	11.5	18.6	198.4	4.7
**YOLOv8-Pose M 640 280,** Filtered to 280 videos (≤2 bad frames)	YOLOv8-Pose M	–	640×640	–	–	–	2.1	6.7	8.6	11	17.6	153.1	4.3
**Full-Frame V-SwinT-B + FPN 224 Aug MSE FPN1024**	V-SwinT-B	2-level features (1024 output channels)	224×224	Yes	MSE	–	8.4	12	13.4	15.4	20.5	38.6	3.4
**V-SwinT-S + FPN 168,** 28 frame input	V-SwinT-S	2-level features (512 output channels)	168×168	No	MSE	–	8.1	12.1	13.7	15.9	21.6	47.1	3.8
**Full-Frame V-SwinT-S + FPN 168 Aug MSE FPN1024**	V-SwinT-S	2-level features (1024 output channels)	168×168	Yes	MSE	–	8.6	12.9	14.8	17.2	23.6	44.1	4.3

Min W./Max W. = whisker values (most extreme non-outliers) Min Out./Max Out. = most extreme points beyond 1.5 × IQR Q1 / Median / Q3 = 25th/50th/75th percentiles. IQR = Q3 – Q1

Figure EXP2-1

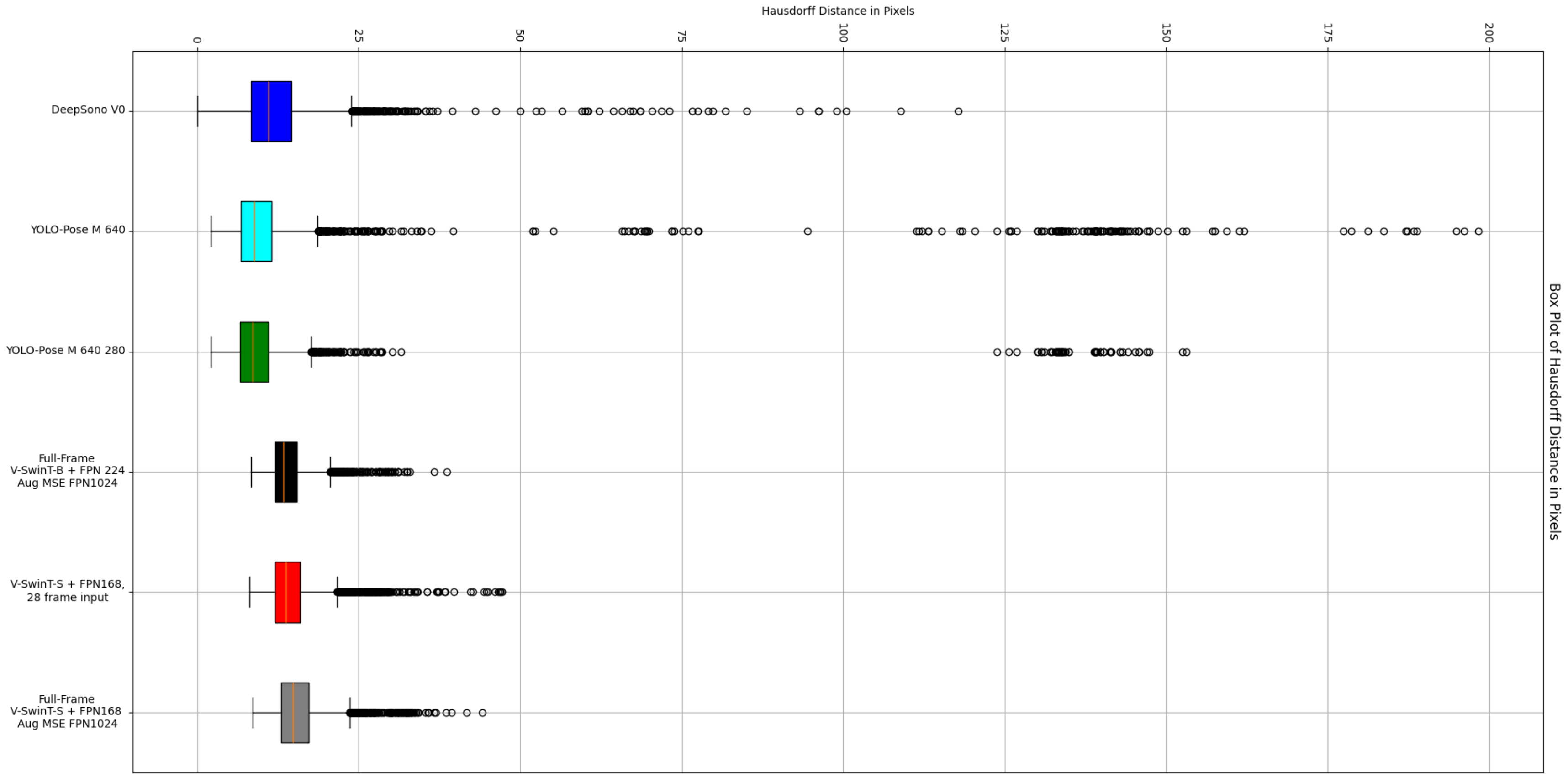

Dice Results of Experiment S2

Table EXP2-2

Model	Backbone	FPN	Input Size	Augmentation	Loss	Dice Min Out.	Dice Min W.	Q1	Median	Q3	Dice Max W.	Dice Max Out.	IQR
**DeepSono V0**	IDOL	–	384×384	–	–	0	0.839	0.902	0.926	0.945	0.993	–	0.043
**YOLOv8-Pose M 640**	YOLOv8-Pose M	–	640×640	–	–	0	0.885	0.931	0.949	0.962	0.988	–	0.031
**YOLOv8-Pose M 640 280,** Filtered to 280 videos (≤2 bad frames)	YOLOv8-Pose M	–	640×640	–	–	0	0.89	0.934	0.951	0.963	0.988	–	0.029
**Full-Frame V-SwinT-B + FPN 224 Aug MSE FPN1024**	V-SwinT-B	2-level features (1024 output channels)	224×224	Yes	MSE	0.791	0.891	0.934	0.951	0.962	0.985	–	0.028
**V-SwinT-S + FPN 168,** 28 frame input	V-SwinT-S	2-level features (512 output channels)	168×168	No	MSE	0.593	0.879	0.926	0.945	0.958	0.985	–	0.032
**Full-Frame V-SwinT-S + FPN 168 Aug MSE FPN1024**	V-SwinT-S	2-level features (1024 output channels)	168×168	Yes	MSE	0.684	0.866	0.918	0.938	0.952	0.984	–	0.034

- **Min W./Max W.** = whisker values (most extreme non-outliers) **Min Out./Max Out.** = most extreme points beyond 1.5 × IQR **Q1 / Median / Q3** = 25th/50th/75th percentiles. **IQR** = Q3 – Q1

Figure EXP2-2

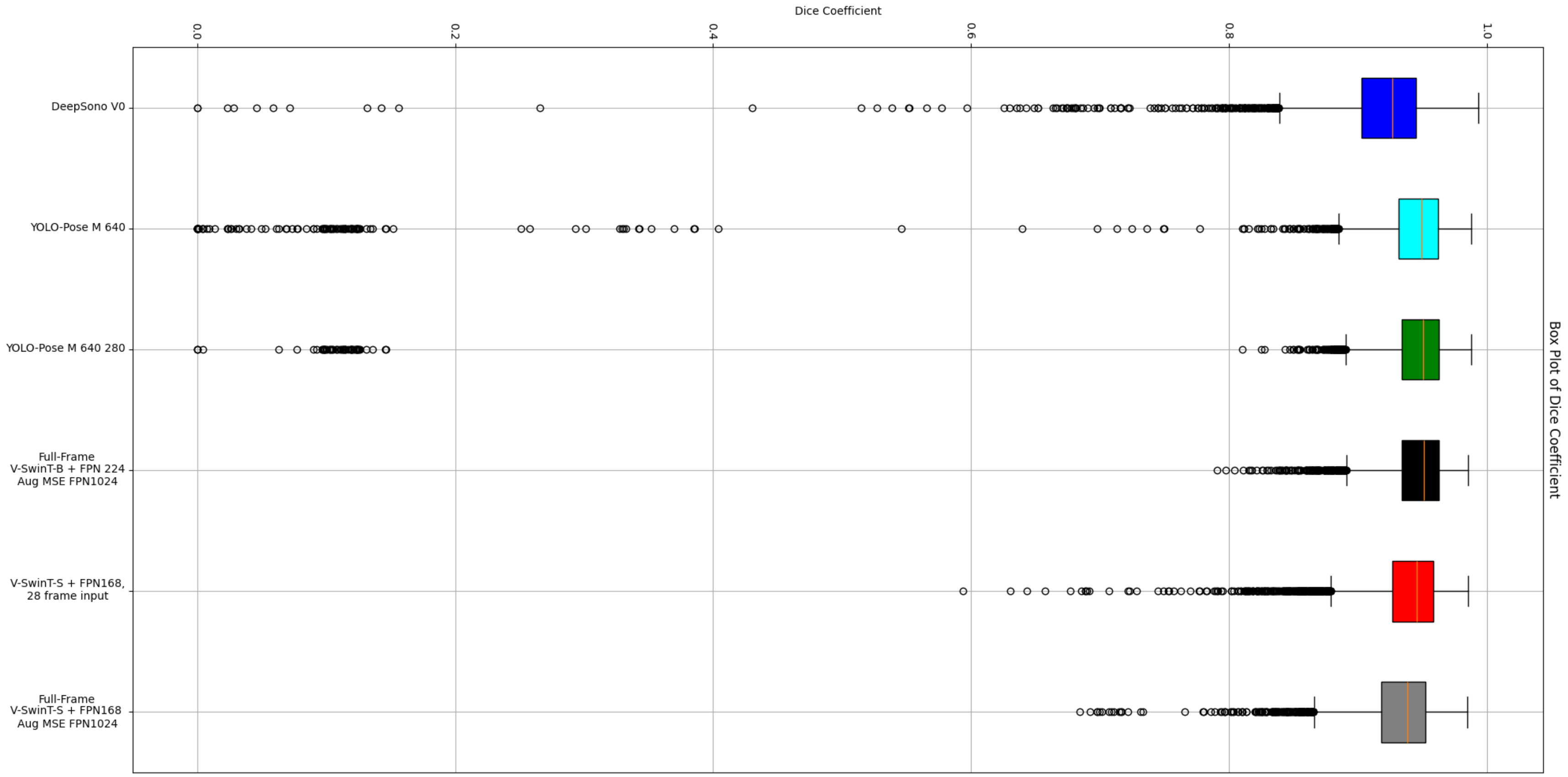

Model differences in a nutshell

- **DeepSono V0 (IDOL)** The baseline "In Defense of Online Learning" (IDOL) architecture employed in our previous model first predicts the left-ventricular myocardium mask, from which the endocardial contour is then extracted.
- **YOLOv8-Pose M** The frame-based models employ a 640×640 YOLOv8-Pose M network to predict the LV endocardial contour point coordinates on a per-frame basis. The "280" variant further excludes any video containing more than two frames with erroneous predictions, yielding a filtered set of 280 videos. Each frame is processed independently, with the network directly outputting the (x, y) coordinates that delineate the left-ventricular endocardial boundary.
- **V-SwinT-S, V-SwinT-B** backbones leverage spatiotemporal Windowed MSA (Video Swin Transformer) to embed temporal context directly at the token level.
- **FPN** variants fuse two layers of multi-scale features before the regression head, improving robustness to scale.
- **FPN output dimensions**: two-level FPN that fuses multi-scale feature maps into a single feature map with **512 , 1024 channels** (i.e., the output FPN feature map's channel dimension), which is then used to regress the 49 contour points of the target frame.
- **Input size** differences (168 vs. 224) affect spatial resolution
- **Aug** (augmentation) introduces basic geometric transforms(scale/translation/rotaton), brightness/contrast jitter to simulate variability.

Final Model Selecton

We chose the **Full-Frame V-SwinT-B + FPN 224 Aug MSE FPN1024** because it achieves the highest median Dice (≈0.951) and lowest median Hausdorff (≈13.4 pixel) with the tightest IQRs—evidence that its deeper backbone, full-sequence processing, and rich 1024-dim FPN yield both superior and consistent contour accuracy.

Experiment S3: fine-tune of model design

Compared Models

1. **DeepSono V0 (IDOL)**
    - The baseline "In Defense of Online Learning" (IDOL) architecture employed in our previous model first predicts the left-ventricular myocardium mask, from which the endocardial contour is then extracted.
2. **YOLOv8-Pose M (All Videos)**
    - Applies a YOLOv8-Pose M backbone to the full 320-video set. It processes each frame independently, directly outputting the coordinates of the points that delineate the LV endocardial contour.
3. **YOLOv8-Pose M (Filtered)**
    - Excludes any clip containing more than two frames with erroneous LV endocardial predictions, resulting in 280 videos.
4. **Full-Frame V-SwinT-B + FPN3 224 Aug MSE FPN1024 ED5 ES10**
    - V-SwinT-B backbone ingesting 64 frames at 224 × 224 pixels
    - Three-level FPN that fuses three scales into a 1024-channel feature map.
    - Data augmentation on; plain MSE loss with Gaussian ED/ES weighting (5× on ED, 10× on ES)
    - Outputs the full 64-frame contour sequence.
5. **Full-Frame V-SwinT-B + FPN3 224 Aug MSE FPN1024 (ImageNet-22k pretrained) ED5 ES10**
    - Same as (4) but initialized with ImageNet-22k weights.
6. **Full-Frame V-SwinT-B + FPN3 240 Aug MSE FPN1024 (240 × 240 input, ImageNet-22k pretrained) ED5 ES10**
    - Higher spatial resolution (240 × 240 pixels) version of (5).
7. **Full-Frame V-SwinT-B + FPN 224 Aug MSE FPN1024 (2-level features)**
    - V-SwinT-B with a simplified two-level FPN (1024 channels), augmented, MSE loss (no ED/ES weighting), full-sequence regression.

Hausdorff Results of Experiment S3

Table EXP3-1

Model	Backbone	FPN	Input Size(HxW)	Augmentation	ED weighting	ES weighting	Loss	Min Out.	Min W.	Q1	Median	Q3	Max W.	Max Out.	IQR
**DeepSono V0**	IDOL	–	384×384	–	-	-	–	–	0	8.3	11	14.6	23.8	117.8	6.3
**YOLOv8-Pose M 640**	YOLOv8-Pose M	–	640×640	–	-	-	–	–	2.1	6.8	8.9	11.5	18.6	198.4	4.7
**YOLOv8-Pose M 640 280,** Filtered to 280 videos (≤2 bad frames)	YOLOv8-Pose M	–	640×640	–	-	-	–	–	2.1	6.7	8.6	11	17.6	153.1	4.3
**Full-Frame V-SwinT-B + FPN3 224 Aug MSE FPN1024 ED5 ES10**	V-SwinT-B	3-level features (1024 output channels)	224×224	Yes	5x	10x	MSE	–	3	7	9	11.4	17.9	36.8	4.4
**Full-Frame V-SwinT-B + FPN3 224 Aug MSE FPN1024**(imgnet-22k pretrained) **ED5 ES10**	V-SwinT-B	3-level features (1024 output channels)	224×224	Yes	5x	10x	MSE	–	2.9	8.1	10.1	13.1	20.5	35.4	5
**Full-Frame V-SwinT-B + FPN3 240 Aug MSE FPN1024**(240×240 input, imgnet-22k pretrained) **ED5 ES10**	V-SwinT-B	3-level features (1024 output channels)	240×240	Yes	5x	10x	MSE	–	3.2	7.3	9.2	11.8	18.6	43.3	4.5
**Full-Frame V-SwinT-B + FPN 224 Aug MSE FPN1024**	V-SwinT-B	2-level features (1024 output channels)	224×224	Yes	-	-	MSE	–	8.4	12	13.4	15.4	20.5	38.6	3.4

Figure EXP3-1

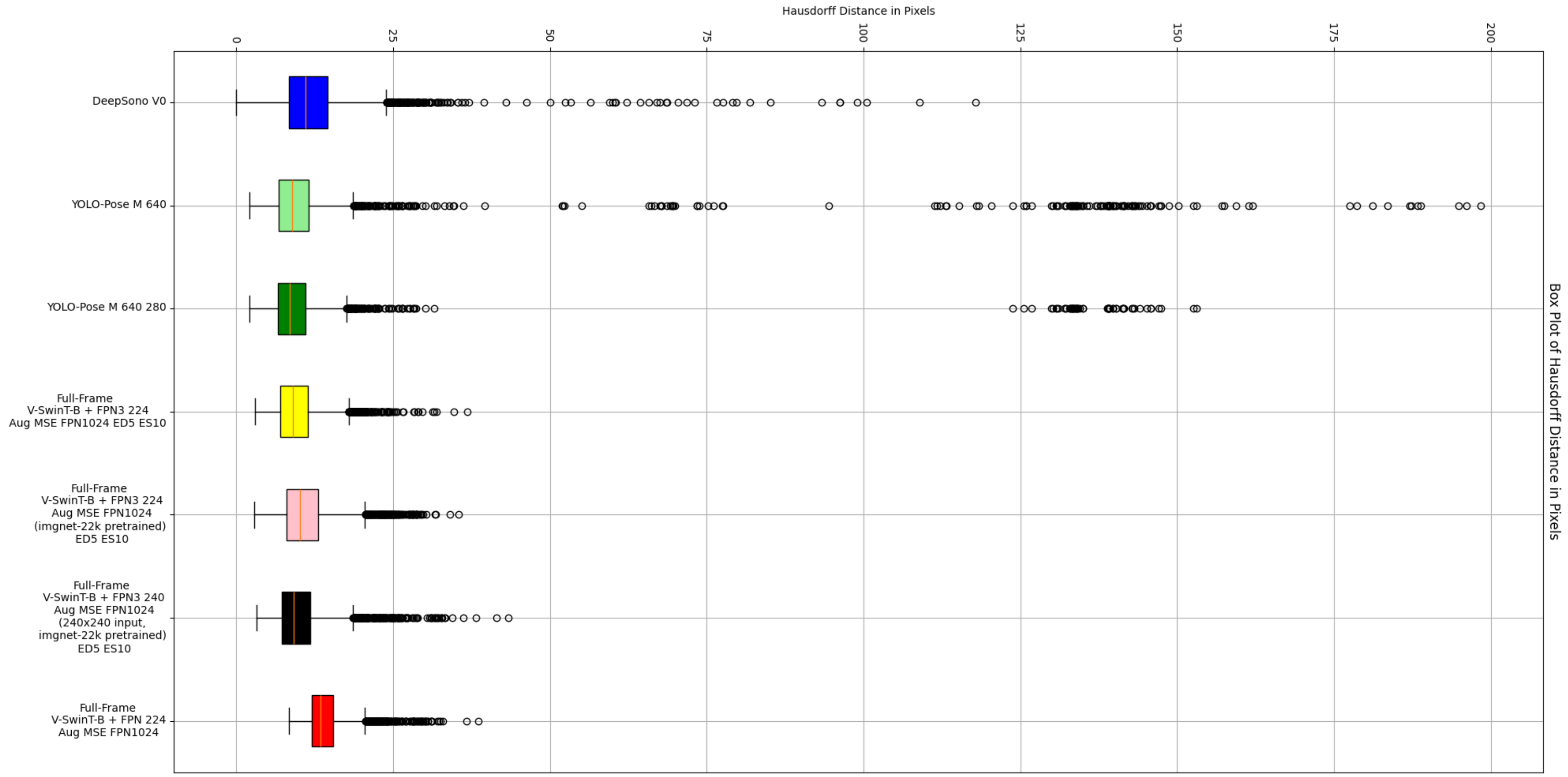

Dice Results of Experiment S3

Table EXP3-2

Model	Backbone	FPN	Input Size	Augmentation	ED weighting	ES weighting	Loss	Dice Min Out.	Dice Min W.	Q1	Median	Q3	Dice Max W.	Dice Max Out.	IQR
**DeepSono V0**	IDOL	–	384×384	–	-	-	–	0	0.839	0.902	0.926	0.945	0.993	–	0.043
**YOLOv8-Pose M 640**	YOLOv8-Pose M	–	640×640	–	-	-	–	0	0.885	0.931	0.949	0.962	0.988	–	0.031
**YOLOv8-Pose M 640 280 ,** Filtered to 280 videos (≤2 bad frames)	YOLOv8-Pose M	–	640×640	–	-	-	–	0	0.89	0.934	0.951	0.963	0.988	–	0.029
**Full-Frame V-SwinT-B + FPN3 224 Aug MSE FPN1024 ED5 ES10**	V-SwinT-B	3-level features (1024 output channels)	224×224	Yes	5x	10x	MSE	0.802	0.892	0.932	0.947	0.958	0.989	–	0.026
**Full-Frame V-SwinT-B + FPN3 224 Aug MSE FPN1024**(imgnet-22k pretrained) **ED5 ES10**	V-SwinT-B	3-level features (1024 output channels)	224×224	Yes	5x	10x	MSE	0.802	0.886	0.927	0.942	0.954	0.98	–	0.027
**Full-Frame V-SwinT-B + FPN3 240 Aug MSE FPN1024**(240×240 input, imgnet-22k pretrained) **ED5 ES10**	V-SwinT-B	3-level features (1024 output channels)	240×240	Yes	5x	10x	MSE	0.749	0.898	0.937	0.952	0.964	0.987	–	0.027
**Full-Frame V-SwinT-B + FPN 224 Aug MSE FPN1024**	V-SwinT-B	2-level features (1024 output channels)	224×224	Yes	-	-	MSE	0.791	0.891	0.934	0.951	0.962	0.985	–	0.028

Figure EXP3-2

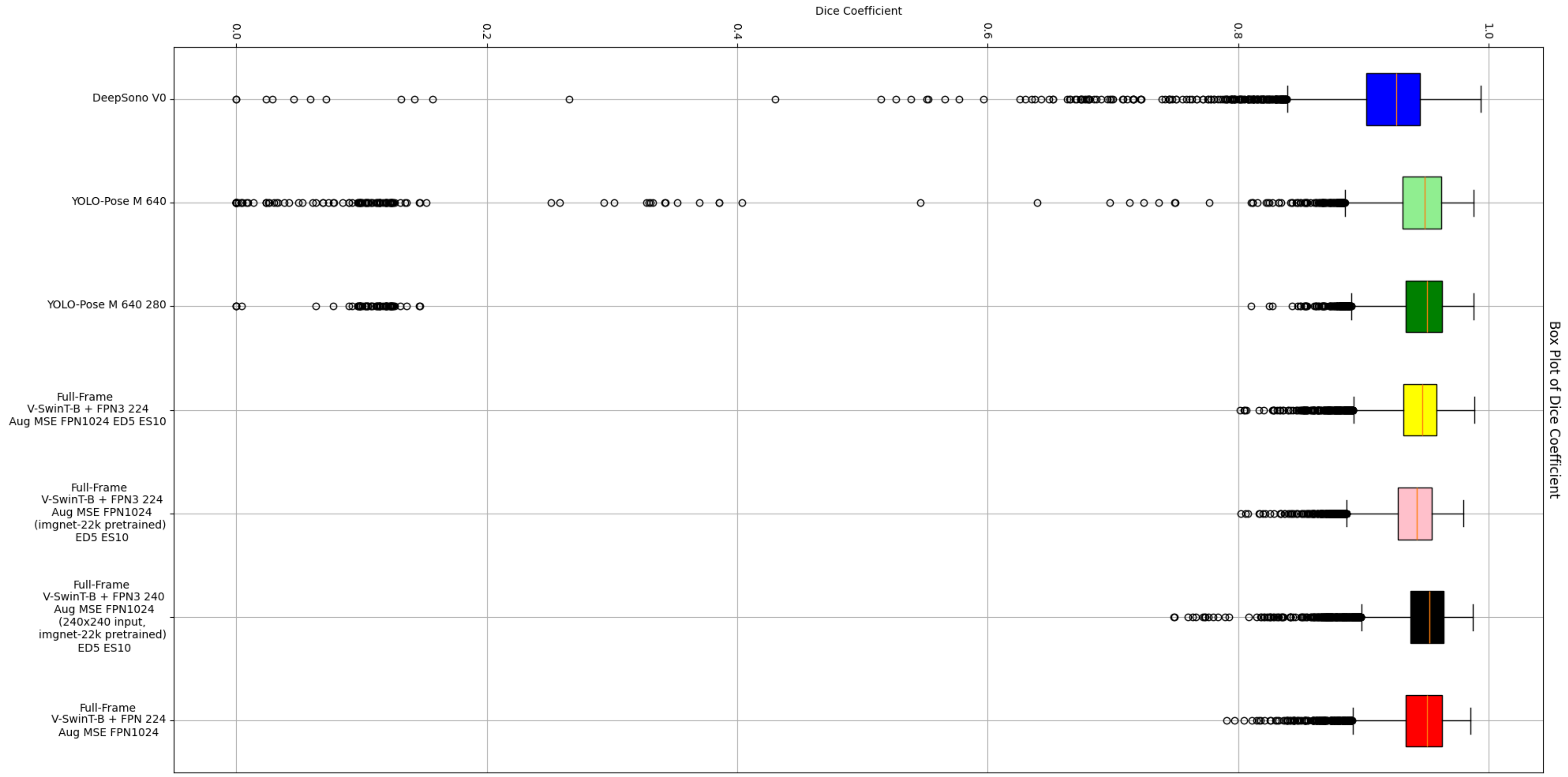

Model differences in a nutshell

- **DeepSono V0 (IDOL)** The baseline “In Defense of Online Learning” (IDOL) architecture employed in our previous model first predicts the left-ventricular myocardium mask, from which the endocardial contour is then extracted.
- **YOLOv8-Pose M** The frame-based models employ a 640 × 640 YOLOv8-Pose M network to predict the LV endocardial contour point coordinates on a per-frame basis. The “280” variant further excludes any video containing more than two frames with erroneous predictions, yielding a filtered set of 280 videos. Each frame is processed independently, with the network directly outputting the (x, y) coordinates that delineate the left-ventricular endocardial boundary.
- **V-SwinT-B** backbones leverage spatiotemporal Windowed MSA (Video Swin Transformer) to embed temporal context directly at the token level.
- **FPN** variants fuse two or three layers of multi-scale features before the regression head, improving robustness to scale.
- **FPN output dimensions**: Three-level FPN that fuses multi-scale feature maps into a single feature map with 1024 **channels** (i.e., the output FPN feature map’s channel dimension), which is then used to regress the 49 contour points of the target frame.
- **Imagenet-22k pretrained weight**: This backbone is initialized with ImageNet-22k pre-trained weights (14 M images, 22 k classes). These 2D parameters are “inflated” into the 3D windowed attention blocks, providing strong spatial priors that could improve contour accuracy when fine-tuning on ultrasound sequences
- **Input size** affects spatial resolution
- **Aug (augmentation)** introduces basic geometric transforms(scale/translation/rotation), brightness/contrast jitter to simulate variability,
- **ED weighting:** We apply a Gaussian temporal weighting window spanning five frames centered on the end-diastole (ED) frame, with a peak weight of 5 at the ED frame, to emphasize diastolic contour accuracy in the loss.
- **ES weighting:** We likewise use a Gaussian window of five frames centered on the end-systole (ES) frame, peaking at weight 5, to prioritize systolic contour accuracy.
- **Input size** affect spatial resolution

Final Model Selecton

ED/ES loss weighting: Applying Gaussian weights around ED and ES frames did **not** yield visible improvements in Dice or Hausdorff metrics—likely a consequence of our relatively small training and test sets.

ImageNet-22k pretraining: Although it provided no gains here, we retain it because the original Video Swin Transformer paper reports significant benefits; on a larger dataset, these pretrained spatial priors may prove more impactful.

FPN depth (three-level vs. two-level): We compared two-level (1024 channels) and three-level (1024 channels) FPN variants. The three-level design did show a clear advantage over two-level fusion in the Hausdorff distance metric, indicating improved boundary accuracy. This suggests that the additional level helps capture finer spatial details, reducing large contour errors.

240 × 240 input resolution: This configuration achieved the highest accuracy but required roughly double the training time, making it impractical for iterative experimentation.

Final selection: We choose **Full-Frame V-SwinT-B + FPN3 224 Aug MSE FPN1024** (imgnet-22k pretrained) **ED5 ES10** as our primary variant. It strikes the best balance—high median Dice, low median Hausdorff, tight IQRs, and reasonable training cost—while aligning with proven transformer pretraining and multi-scale feature principles.

Dynamic-Programming ED–ES (DP-EDES) Algorithm

1 Input: sequence of contour curves

For every frame $t = 1, \dots, T$ the segmentation network (or an earlier post-processor) supplies a **closed poly-line**

$$C_t = \{(x_{t,1}, y_{t,1}), (x_{t,2}, y_{t,2}), \dots, (x_{t,N_t}, y_{t,N_t})\}, (x_{t,N_t+1}, y_{t,N_t+1}) = (x_{t,1}, y_{t,1}).$$

The **contour-length signal** is obtained by summing successive Euclidean chord lengths:

$$L_t = \sum_{i=1}^{N_t} \sqrt{(x_{t,i+1} - x_{t,i})^2 + (y_{t,i+1} - y_{t,i})^2}$$

Because the LV fills during diastole and contracts during systole, L_t rises then falls in each heartbeat, making it a reliable 1-D surrogate for phase tracking.

2 Latent-state model

Symbol	Definition
$s_t \in \{+1, -1\}$	+1 = diastole, −1 = systole
$\Delta_t = L_t - L_{t-1}$	Signed frame-to-frame length change
$\mathbf{1}(\cdot)$	Iverson indicator (1 if true, 0 otherwise)

3 Data-adaptive switching penalty

$$Z = \sqrt{\left(\frac{1}{T} \sum_{t=1}^{T} (L_t - \bar{L})^2\right)}, \qquad \bar{L} = \frac{1}{T} \sum_{t=1}^{T} L_t$$

Using the **standard deviation of the raw length trace** means **no manual tuning**, it automatically adapts to the input signal’s range of variation, and is large enough to suppress most noisy jitters.

4 Optimisation objective

$$J(s_{1:T}) = \sum_{t=2}^{T} [-\Delta_t \, s_t + Z \cdot \mathbf{1}(s_t \neq s_{t-1})] \qquad \text{(DP.1)}$$

Instantaneous term $-\Delta_t s_t$ rewards monotonically **rising** L_t in diastole and **falling** L_t in systole. *Switch term* Z penalises each phase change yet imposes **no cycle-length prior**, so arrhythmic or variable-rate beats are allowed.

5 Dynamic-programming solution

Initialize $F_1(+1) = F_1(-1) = 0$.
For $t \geq 2$

$$F_t(s) = -\Delta_t\, s + \min_{s' \in \{+1,-1\}} [F_{t-1}(s') + Z \cdot \mathbf{1}(s_t \neq s_{t-1})], \quad \begin{matrix} s : current\ state \\ s' : previous\ state \end{matrix} \qquad \text{(DP.2)}$$

and store back-pointers $b_t(s)$ giving the arg-min.
Complexity: $O(2T)$ time, $O(2T)$ memory.

6. Physiological "constraints" conditions (physiological range of the cardiac cycle)

An optional **physiological run-length prior** can be imposed **inside** the DP—without any post-processing—by expanding each state to

$$(\mathrm{s_t}, \mathrm{r_t}), \ \mathrm{s_t} \in \{+1, -1\}, \ \mathrm{r_t} \in \{1,2, \ldots, R_{max}\},$$

where r_t is the number of consecutive frames spent in the current phase.
A switch from s_t to $-s_t$ is allowed only when the preceding run length satisfies

$$R_{min} \leq \ r_t \leq R_{max}, with \ R_{min} = \lfloor f_{fps}\, T_{min} \rfloor, R_{max} = \lceil f_{fps}\, T_{max} \rceil$$

Typical values (clinical range ≈ 40–200 bpm, i.e. $T_{\min} = 0.30$ s, $T_{\max} = 1.50$ s) are:

Frame-rate f_{fps}	$R_{\min}$	$R_{\max}$
25 fps	$\lfloor 25 \times 0.30 \rfloor = 7$ frames	$\lceil 25 \times 1.50 \rceil = 38$ frames
60 fps (high-speed)	$\lfloor 60 \times 0.30 \rfloor = 18$ frames	$\lceil 60 \times 1.50 \rceil = 90$ frames

Embedding these bounds enlarges the DP lattice by a factor of $R_{\max}$ (e.g., ≤ 90 at 60 fps), which remains computationally modest while guaranteeing that every accepted beat lies within the physiological heart-rate window. Users can therefore toggle between the minimalist two-state model and this constrained (s, r) model depending on data quality, with no separate filtering stage required.

Complexity: $O(2R_{\max}T)$ time, $O(2R_{\max}T)$ memory.

7 Key advantages

- **Globally optimal**, **linear-time** ED/ES labelling.
- Only one hyper-parameter **Z**, automatically derived from the input trace.
- **No assumption on heartbeat length** → handles arrhythmias, pauses, tachycardia.
- Robust to speckle noise and minor contour-tracking jitter.

Application of DTHR-SegStrain to A2C, A3C and averaged GLS

In the 1,050-case validation cohort, 2 cases lacked the A2C view and 9 lacked the A3C view, resulting in 1,040 cases with all three standard views available for averaged GLS calculation. The DTHR-SegStrain model successfully performed strain estimation across all available views. In contrast, the Unity model successfully processed 1,015 cases in A2C, 1,014 in A3C, and only 1,007 in A4C, allowing averaged GLS computation in just 947 cases. Data are summarized in Table S1 and Figure S1-2.

We evaluated the agreement between AI-derived GLS and expert reference values across individual views (A2C, A3C) and the averaged 3-view GLS (3GLS). In the A2C view, DTHR-SegStrain achieved a Spearman's ρ of 0.8450 and an ICC of 0.83, with a mean bias of 0.488% and 95% limits of agreement (LOA) of ±5.61%. In contrast, Unity showed weaker correlation (ρ = 0.7575, ICC = 0.69), a larger negative bias of –1.63%, and substantially wider LOA (±8.85%).

In the A3C view, DTHR-SegStrain again outperformed Unity, yielding a higher correlation (ρ = 0.8585 vs. 0.7852), a comparable ICC (0.74 vs. 0.74), and a slightly smaller LOA (±5.11% vs. ±7.68%). But the absolute bias was larger for DTHR-SegStrain (1.143%) than for Unity (0.351%).

Most notably, when averaging GLS across A2C, A3C, and A4C views, DTHR-SegStrain achieved the highest consistency with expert reference (ρ = 0.9193, ICC = 0.91), with the narrowest LOA (±3.51%) and a minimal bias of –0.21%. In comparison, Unity's averaged GLS yielded a lower correlation (ρ = 0.8834), ICC of 0.85, a larger bias of –0.948%, and wider LOA (±5.40%).

Table S1

Method	View	Expert strain	AI strain	ρ	ICC (mean, CI)	Bland-Altman analysis	
						Mean difference, CI	LOA
DTHR-SegStrain<br><br>N=1040	A2C	-16.5±5.0	-16.0±4.4	0.8450	0.833<br>(0.814-0.851)	0.488<br>(0.317 to 0.658)	±5.605
	A3C	-16.4±5.0	-15.3±4.4	0.8585	0.853<br>(0.836-0.896)	1.143<br>(0.988 to 1.299)	±5.106
	A4C	-16.6±5.3	-17.6±5.1	0.8868	0.884<br>(0.871-0.897)	-1<br>(-1.152 to -0.847)	±5.014
	Avg	-16.5±4.9	-16.3±4.5	0.9193	0.913<br>(0.903-0.923)	-0.21<br>(0.091 to 0.330)	±3.509
Unity<br><br>N=947	A2C	-16.6±5.0	-18.2±6.3	0.7575	0.695<br>(0.660-0.726)	-1.63<br>(CI -1.912 to -1.348)	±8.854
	A3C	-16.5±5.0	-16.8±5.8	0.7852	0.747<br>(0.718-0.774)	-0.351<br>(CI -0.596 to -0.106)	±7.684
	A4C	-16.6±5.3	-17.5±5.8	0.8153	0.690<br>(0.655-0.722)	-0.863<br>(CI -1.141 to -0.584)	±8.735
	Avg	-16.6±5.0	17.5±5.1	0.8834	0.854<br>(0.836-0.870)	-0.948<br>(CI -1.120 to -0.776)	±5.398

A2C: apical two chambers view; A3C: apical three chambers view; A4C: apical four chambers view; Avg: averaged strain values from A2C, A3C and A4C, representing the global longitudinal strain; CI: 95% confidence interval; ICC: Intraclass Correlation Coefficient; LOA: Limits of Agreement; ρ: Spearman's coefficient

Figure S1

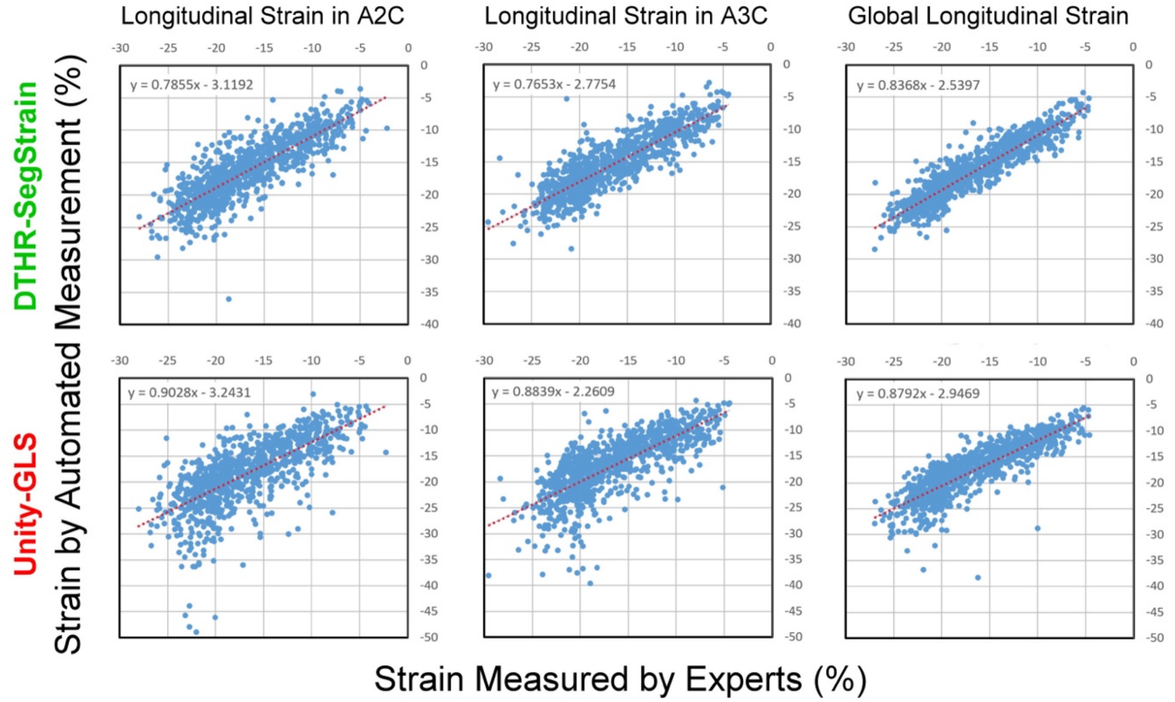

Longitudinal Strain in A2C
Longitudinal Strain in A3C
Global Longitudinal Strain
DTHR-SegStrain
Unity-GLS
Strain by Automated Measurement (%)
y = 0.7855x - 3.1192
y = 0.7653x - 2.7754
y = 0.8368x - 2.5397
y = 0.9028x - 3.2431
y = 0.8839x - 2.2609
y = 0.8792x - 2.9469
Strain Measured by Experts (%)

Figure S2

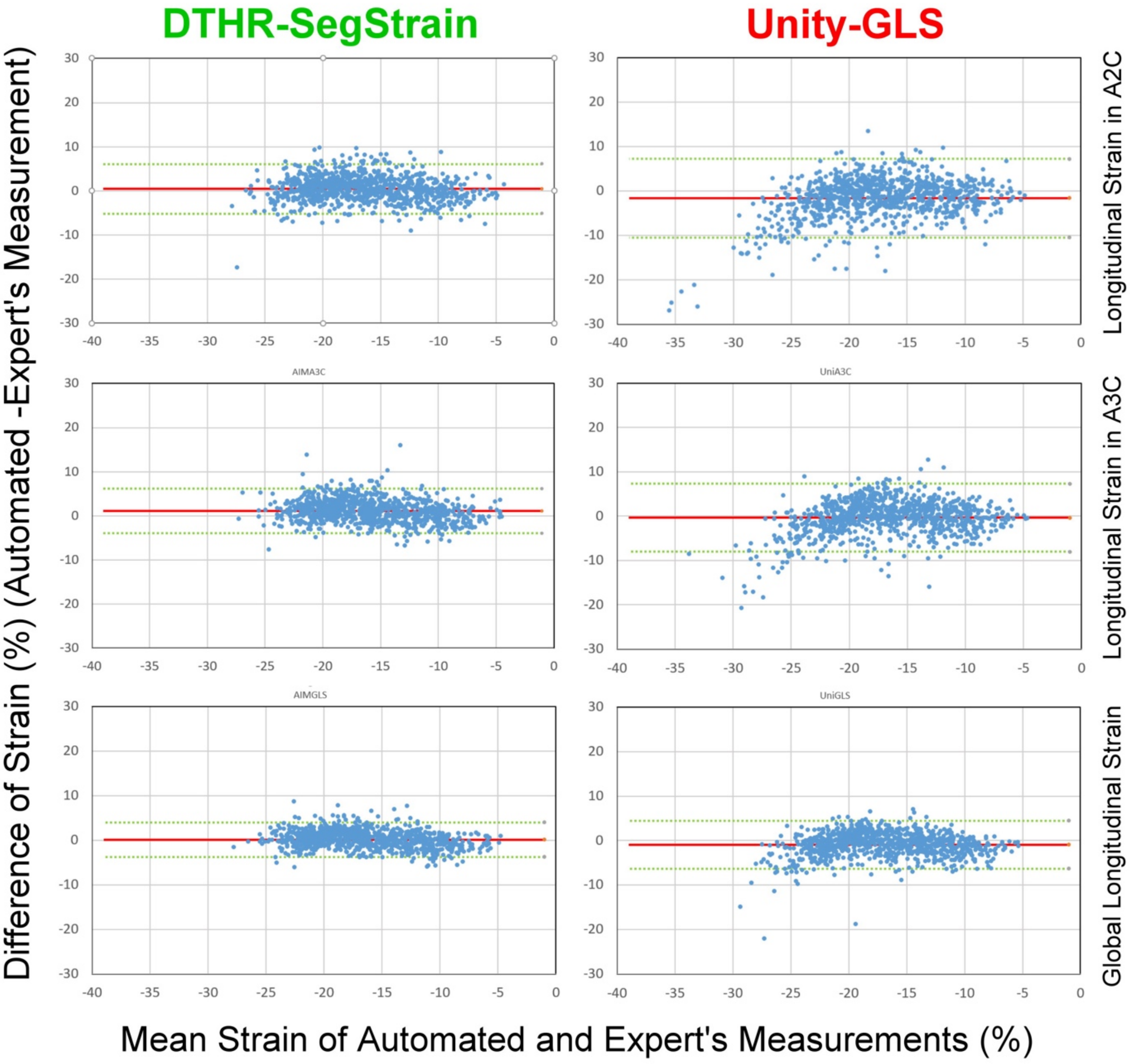

DTHR-SegStrain
Unity-GLS
Difference of Strain (%) (Automated -Expert's Measurement)
Longitudinal Strain in A2C
Longitudinal Strain in A3C
Global Longitudinal Strain
AIMA3C
UniA3C
AIMGLS
UniGLS
Mean Strain of Automated and Expert's Measurements (%)
